# Dimension reduction of dynamics on modular and heterogeneous directed networks

**DOI:** 10.1093/pnasnexus/pgad150

**Published:** 2023-05-02

**Authors:** Marina Vegué, Vincent Thibeault, Patrick Desrosiers, Antoine Allard

**Affiliations:** Département de physique, de génie physique et d’optique, Université Laval, 2325 rue de l’Université, G1V 0A6 Québec, Canada; Centre interdisciplinaire en modélisation mathématique, Université Laval, 2325 rue de l’Université, G1V 0A6 Québec, Canada; Département de physique, de génie physique et d’optique, Université Laval, 2325 rue de l’Université, G1V 0A6 Québec, Canada; Centre interdisciplinaire en modélisation mathématique, Université Laval, 2325 rue de l’Université, G1V 0A6 Québec, Canada; Département de physique, de génie physique et d’optique, Université Laval, 2325 rue de l’Université, G1V 0A6 Québec, Canada; Centre interdisciplinaire en modélisation mathématique, Université Laval, 2325 rue de l’Université, G1V 0A6 Québec, Canada; CERVO Brain Research Center, 2301 avenue d’Estimauville, G1E 1T2 Québec, Canada; Département de physique, de génie physique et d’optique, Université Laval, 2325 rue de l’Université, G1V 0A6 Québec, Canada; Centre interdisciplinaire en modélisation mathématique, Université Laval, 2325 rue de l’Université, G1V 0A6 Québec, Canada

**Keywords:** dimension reduction, networks, nonlinear dynamics, community structure, spectral decomposition

## Abstract

Dimension reduction is a common strategy to study nonlinear dynamical systems composed by a large number of variables. The goal is to find a smaller version of the system whose time evolution is easier to predict while preserving some of the key dynamical features of the original system. Finding such a reduced representation for complex systems is, however, a difficult task. We address this problem for dynamics on weighted directed networks, with special emphasis on modular and heterogeneous networks. We propose a two-step dimension-reduction method that takes into account the properties of the adjacency matrix. First, units are partitioned into groups of similar connectivity profiles. Each group is associated to an observable that is a weighted average of the nodes’ activities within the group. Second, we derive a set of equations that must be fulfilled for these observables to properly represent the original system’s behavior, together with a method for approximately solving them. The result is a reduced adjacency matrix and an approximate system of ODEs for the observables’ evolution. We show that the reduced system can be used to predict some characteristic features of the complete dynamics for different types of connectivity structures, both synthetic and derived from real data, including neuronal, ecological, and social networks. Our formalism opens a way to a systematic comparison of the effect of various structural properties on the overall network dynamics. It can thus help to identify the main structural driving forces guiding the evolution of dynamical processes on networks.

**Significance Statement**

Brains, ecosystems, and populations are all examples of complex systems whose global, collective behaviors are the consequence of the local interactions between their constituting elements (e.g. neurons, species, individuals). The sheer size of these systems as well as their intrinsic heterogeneity make them difficult to study through mathematical models. We develop a principled and systematic method to reduce the number of dimensions of mathematical models of various complex systems, thus bringing them at a scale that greatly facilitates their analysis. We demonstrate the accuracy of our approach using synthetic as well as empirical complex networks, thereby illustrating how it sheds a systematic light on the impact of the different levels of coarse-graining on which several mathematical models rely.

## Introduction

Dimension-reduction methods seek to find a low-dimensional representation of a high-dimensional system. This representation should preserve various key properties of the original system while being more amenable to analysis in order to provide insights on the inner workings and the long-term behavior of the system. The dimension of a proper reduction can also inform on the effective dimension of the original system, namely the extent to which it can be compressed into a simpler form. These reasons make dimension reduction not only necessary for practical or computational purposes but also interesting from a purely theoretical standpoint.

Dimension-reduction methods appear in many areas of science and under different names. For example, the ubiquity of high-dimensional data and the difficulty of extracting the relevant patterns have made dimension reduction an essential problem in data analysis (1–3). Dimension reduction is also relevant in the context of control systems, engineering, and fluid mechanics, where it is referred to as *model order reduction* (4–6) and where the focus is often on linear systems. Methods to reduce the dimension of systems of ordinary differential equations (ODEs) have a relatively long tradition in chemistry, where they are often known as *lumping* (7–9). Yet, these latter works focus on the kinetic theory of molecular reactions and the methods are often applied to dynamics involving only a small number of molecules.

The complexity inherent to large, nonlinear dynamical systems of interacting units (ecological communities, neuronal networks, etc.) makes them very difficult to study.

These systems often exhibit emergent collective phenomena (pandemics, synchronization, etc.) (10–12) and may undergo phase transitions and bifurcations as a perturbation is introduced. Despite often having a huge, qualitative impact on their collective behavior, these transitions and the parameter values at which they appear (the *tipping points*) tend to be very hard to predict (13, 14). One of the major challenges of network science is to find ways to approximate these systems by ones of reduced dimension so as to make them more tractable analytically and computationally and thus to make their tipping points easier to anticipate. In a world threatened by a potential ecological collapse and by the emergence of new pandemics, this task has become more necessary than ever.

How to construct a reduced version of a generic complex system is still an open question (15). Dimension reduction is especially challenging when the original system is highly heterogeneous, i.e. when the rules that define the units’ dynamics and their interactions vary largely across the different units. A standard way to model the influence that each unit (or node) has on the activity of the others is by means of a weighted adjacency matrix plus a set of coupling functions of the nodes’ activities (16, 17). The adjacency matrix can be either a constant of the system or change in time depending on the nodes’ activity, as in plastic neuronal networks (18, 19). According to this framework, a heterogeneous system is a system in which either the adjacency matrix or the functions that define the dynamics exhibit a large variability across the different nodes.

Several dimension-reduction strategies for dynamics on networks have been proposed in recent years. Gao et al. (20) developed a method for reducing an *N*-dimensional system to a one-dimensional one that approximately models the temporal evolution of an effective activity variable. This variable is an average of the nodes’ activities, weighted by their outgoing degrees. This approach was used to predict the resilience of different real-world networks under several types of perturbations. Jiang et al. (21) defined a strategy for predicting the resilience and tipping points of bipartite plant–pollinator networks in which the reduced system is two-dimensional. The two variables in the reduced dynamics then correspond to the (weighted) average abundance of the plants and pollinators, respectively. Recently, Tu et al. (22) proposed a dimension reduction framework designed for systems that are heterogeneous in terms of the functions that define the self-dynamics and the coupling-dynamics of the nodes. The reduced dynamics in this case is one-dimensional and incorporates a variable number of control parameters.

Although one- and two-dimensional reductions have been proven to be effective in some cases, the large heterogeneity of some real-world networks (23) and their tendency to form intricate community structures (24, 25) make them unlikely to be well understood by means of systems of such a low dimension. References (26, 27) introduced formalisms for reducing an *N*-dimensional system to a system of arbitrary dimension n<N. The *n* variables of the reduced system are constructed by taking into account the spectral properties of the adjacency matrix (as well as the matrix of self-coupling dynamical parameters in Ref. (27)), regardless of the specific form of the coupling functions. The reduction method defined in Ref. (26) is appropriate for undirected networks with weakly coupled communities or with a bipartite structure. Reference (27) generalized the formalism to dynamical systems with heterogeneous dynamical parameters on generic undirected networks, including the ones with strongly coupled modules. Both approaches have been tested on undirected networks with homogeneous group connectivity, which is also a very common structural assumption to study synchronization of coupled oscillators through dimension reduction (28–32). It remained unclear, however, how these approaches could be adapted to reduce the dimension of dynamics on directed networks with significant heterogeneous connectivity.

**Fig. 1.**
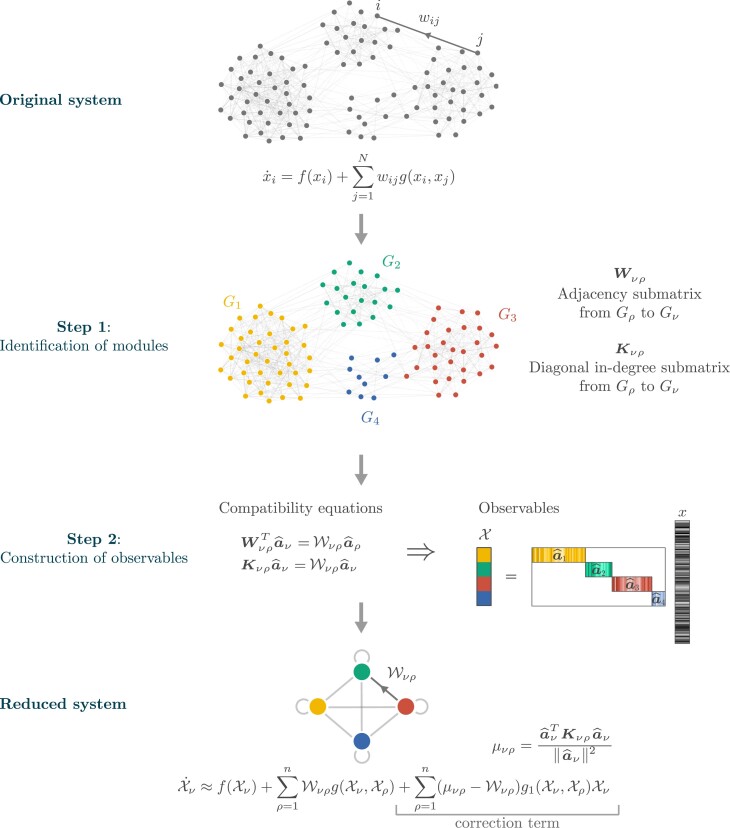
Method schematics of the spectral reduction. First, the nodes are partitioned into assortative or disassortative groups so that nodes in the same group share similar connectivity properties. In this case, n=4 groups were defined. Group-to-group adjacency and in-degree submatrices are defined from the original adjacency matrix. Second, these matrices are used to solve the compatibility equations on the partial reduction vectors ${\hat{a}}_{1},\ldots ,{\hat{a}}_{n}$ and the matrix $W=(W_{\nu \rho }{)}_{\nu ,\rho =1}^{n}$. Once the compatibility equations are solved, the observables are constructed as the scalar product of the reduction vectors and the activity vector x. The final approximate reduced dynamics on the observables is analogous to the original dynamics—with W acting as a reduced adjacency matrix—and can also incorporate a correction term.

We develop a dimension-reduction method for dynamical systems on directed networks that are possibly modular (i.e. that can contain groups of nodes with similar connectivity properties, the *modules*) but whose modules are significantly heterogeneous. We thus discard all the structural limitations that were previously imposed on networks. The method is defined by a two-step process illustrated in Fig. 1. First, the nodes are classified into *n* groups of nodes that share similar connectivity properties (these could correspond to the modules, or to subgroups within the modules, in the case of modular networks). The variables of the reduced system, the *observables*, are weighted averages of the node activities within each group. This means that the number of groups in the node partition, *n*, corresponds to the dimension of the reduced system. We refer to the vectors that specify these group averages as the *reduction vectors*. Generically, the observables’ dynamics cannot be expressed as a closed system of ODEs: the exact observables’ dynamics is a function of the nodes’ activities but it cannot be expressed as a function of the observables themselves. The second step consists of approximately closing it. For this, we approximate the dynamics’ functions and find conditions on the reduction vectors that make this approximation as accurate as possible. These conditions, that we dub the *compatibility equations*, determine the reduction vectors and the parameters of the reduced system, which define a *reduced adjacency matrix*. The result is a *reduced system* of dimension *n* on a *reduced network*.

The paper is structured as follows. First, we present the dimension-reduction strategy together with a method for approximately solving the compatibility equations. Then, following the common practice in the network science community for assessing the quality of dimension reductions (20–22, 33–36), we test the capacity of our method to predict the bifurcation diagrams of different systems, for various types of dynamics and synthetic network topologies. We finally apply our method to dynamical systems inspired by three types of real-world networks and we show that satisfactory bifurcation diagrams can be produced by tuning the dimension of the reduced system. All the theoretical results used to derive the equations presented in the paper are detailed in the Supplementary material.

## Dimension-reduction strategy

### Overview

In many real-world networks, the nodes can be partitioned into groups of densely connected nodes (commonly referred to as *modules*) (25, 37–39). Usually, a module corresponds to a group of nodes that are also involved in a common task, which suggests that the structural modularity seen in these networks reflects a modularity at the functional level (25, 39). Other networks have groups of sparsely connected nodes whose connectivity profiles with nodes in other groups are similar. For example, in plant–pollinator networks, pollinators interact with plants and plants with pollinators, but it is assumed that plants do not directly interact with other plant species and analogously for pollinators (i.e. the interaction network is bipartite). We can thus say that the set of plants and the set of pollinators constitute two different groups in terms of their connectivity profiles. Either if the structure of these networks tends to be assortative (nodes of similar connectivity properties tend to be connected) or dissortative (nodes of similar connectivity properties tend to be disconnected), they have in common the fact that their nodes can be classified into groups of nodes that share similar connectivity properties. Throughout this paper, we use the terms *modularity* and *community structure* to refer to this network property, regardless of whether the groups are densely or sparsely connected, and *modules* or *communities* to refer to these groups.

We present a strategy to reduce the dimension of a dynamical system taking into account the community structure of the interaction network. We assume that the *N* nodes of the original system interact by means of a not necessarily symmetric adjacency matrix $W=(w_{ij}{)}_{i,j}\in R^{N\times N}$ and that their activities evolve in time according to a dynamics of the form


(1) $${\dot{x}}_{i}=f(x_{i})+\sum_{\;j=1}^{N}w_{ij}g(x_{i},x_{j}),$$

with i∈{1,…,N}, and where $x_{i}$ is a real-valued function of a real variable (time) that represents the activity of node *i*. The functions *f* and *g* are generic functions of class $C^{1}$ (i.e. 1-time differentiable with continuous derivatives). The function *f* defines the self-dynamics of the nodes, while *g* accounts for the dynamical coupling between pairs of nodes. The weight $w_{ij}\in R$ encodes the strength of the interaction from node *j* to node *i*.

This model assumes that node heterogeneity comes from the adjacency matrix itself (the functions *f* and *g* are the same for all the nodes). It is therefore reasonable to assume that nodes with a similar connectivity profile will have similar activities. We aim to construct a reduced version of the system by defining a set of *linear observables*, each of them representing a weighted average of the node activities within each of the connectivity-based communities. Thus, our reduced system has the same dimension as the number of modules in the original network.

Let us suppose that we know the community structure in our network. This means that we have a *partition* of the nodes into groups: each node belongs to one and only one of these groups. We define *n* as the number of groups and denote the groups by $G_{1},\ldots ,G_{n}$ and their corresponding observables by $X_{1},\ldots ,X_{n}$. For all ν∈{1,…,n}, the observable $X_{\nu }$ is a linear combination of the activities of nodes in group $G_{\nu }$: $X_{\nu }$ is defined by a nonnegative, normalized weight vector $a_{\nu }=(a_{\nu i}{)}_{i=1}^{N}\in R^{N}$:


(2) $$X_{\nu }\;:\;=\sum_{i=1}^{N}a_{\nu i}x_{i},\;\sum_{i=1}^{N}a_{\nu i}=1,\;a_{\nu i}=0\text{ if }i\notin G_{\nu }.$$

We call $a_{1},\ldots ,a_{n}$ the *reduction vectors*.

To reduce the dimension of our system, we need to specify how to map the original dynamics (Eq. 1) into a reduced dynamics for the *n* observables. This implies (a) finding what the vectors $a_{1},\ldots a_{n}$ are and (b) providing a system of ODEs for the temporal evolution of the observables.

We provide the details in the following sections. A rigorous derivation of all the results is provided in the Supplementary material.

### Group adjacency and in-degree matrices

Let us denote by $m_{1},\ldots ,m_{n}$ the sizes of the groups in our network:


$$m_{\rho }\;:\;=|G_{\rho }|,\;\rho \in \{1,\ldots ,n\}.$$

Without loss of generality we can suppose that the nodes have been reordered so that the indices of nodes in each group are consecutive numbers:


(3) $$G_{\nu }=\left\{1+\sum_{\rho =1}^{\nu -1}m_{\rho },\ldots ,\sum_{\rho =1}^{\nu }m_{\rho }\right\}.$$

This allows us to express the adjacency matrix W in the block form


(4) $$W=\left(\begin{array}{ccc} W_{11} & \cdots & W_{1n}\\ \vdots & \ddots & \vdots \\ W_{n1} & \cdots & W_{nn} \end{array}\right),$$

where $W_{\nu \rho }$ is the submatrix of size $m_{\nu }\times m_{\rho }$ that includes all the interaction weights from nodes in group $G_{\rho }$ to nodes in group $G_{\nu }$. In a similar way, we can define the group-to-group weighted in-degree diagonal matrix $K_{\nu \rho }=\text{diag}(k_{i_{1}}^{\rho },\ldots ,k_{i_{m_{\nu }}}^{\rho })$, where $\{i_{1},\ldots ,i_{m_{\nu }}\}=G_{\nu }$ and $k_{i}^{\rho }=\sum_{\;j\in G_{\rho }}w_{ij}$ is the weighted in-degree of node *i* only taking into account connections that come from nodes in group $G_{\rho }$. Thus, the global diagonal in-degree matrix can be expressed as a function of the group-to-group in-degree matrices as


(5) $$K=\left(\begin{array}{ccc} K_{11}+\cdots +K_{1n} & \cdots & 0\\ \vdots & \ddots & \vdots \\ 0 & \cdots & K_{n1}+\cdots +K_{nn} \end{array}\right).$$

### Reduction vectors and approximate reduced dynamics

From Eqs. 1 and 2, the exact temporal evolution of a given observable $X_{\nu }$ is


(6) $${\dot{X}}_{\nu }=\sum_{i=1}^{N}a_{\nu i}f(x_{i})+\sum_{i,j=1}^{N}a_{\nu i}w_{ij}g(x_{i},x_{j}).$$

Our objective is to rewrite Eq. 6 in a closed form, that is, to make it be a function of the observables only. This cannot be fulfilled exactly in general, but we can work on Eq. 6 so that it admits an *approximate* closed form.

This is accomplished by following three basic steps:

1. Substitute $f(x_{i})$ and $g(x_{i},x_{j})$ by affine approximations in $x_{i}$ and $x_{j}$ around the observables associated to the groups to which nodes *i* and *j* belong ($X_{\nu }$ and $X_{\rho }$ for $i\in G_{\nu }$ and $j\in G_{\rho }$). In general, the resulting approximations are nonlinear functions of $X_{\nu }$ and $X_{\rho }$.
2. Introduce these approximations in Eq. 6. Find conditions on the reduction vectors which ensure that this approximate system is closed (i.e. that it is expressed as a function of the observables only). These conditions are called the *compatibility equations*. The approximate closed system of ODEs is the reduced system we are looking for.
3. Solve the compatibility equations to find the reduction vectors. These vectors specify the observables and determine the adjacency matrix of the reduced system.

We considered affine approximations because approximations of a larger order would require the use of nonlinear observables (40, Appendix B) and this is beyond the scope of the present work. The justification behind approximating $f(x_{i})$ and $g(x_{i},x_{j})$ around the corresponding observables is the following: if the node partition reflects a true organization of the nodes into groups of similar connectivity profiles, then the nodes within the same group should have similar activities, and these activities should be close to the corresponding observable at any time. The validity of this assumption will of course depend on the properties of the chosen partition, especially on the number of groups and on the inter-node heterogeneity within each group.

We call our reduction the *spectral reduction* for reasons that will become clear below. For the purpose of comparison, we also consider a *homogeneous reduction* that results from taking a zeroth-order Taylor approximation of functions *f* and *g* and whose reduction vectors are homogeneous over the groups.

#### Homogeneous reduction

We here approximate $f(x_{i})\approx f(X_{\nu })$ and $g(x_{i},x_{j})\approx g(X_{\nu },X_{\rho })$ whenever $i\in G_{\nu }$ and $j\in G_{\rho }$, assuming as usual that all conditions in Eq. 2 are satisfied. Doing so immediately transforms Eq. 6 into the following approximate closed form, regardless of the particular choice of reduction vectors $a_{1},\ldots ,a_{n}$:


(7) $${\dot{X}}_{\nu }\approx f(X_{\nu })+\sum_{\rho =1}^{n}W_{\nu \rho }g(X_{\nu },X_{\rho }),$$

where


(8) Wνρ:=∑i∈Gνj∈Gρaνiwij=∑i∈Gνaνikiρ

is the weighted in-degree coming from nodes in $G_{\rho }$, averaged over nodes in $G_{\nu }$ according to $a_{\nu }$ (see Lemma 2 in section “Approximate reduced dynamics” of the Supplementary material for details).

The form of the reduced dynamics (Eq. 7) is analogous to that of the original dynamics. The matrix $W=(W_{\nu \rho }{)}_{\nu ,\rho }$ is the *reduced adjacency matrix*: a weighted matrix of interactions among the observables in the approximate reduced system. Despite the reduced system has this form for any choice of the reduction vectors, the observables’ value at any given time and the reduced adjacency matrix do depend on these vectors, and thus the reduction’s accuracy will depend on them too.

We choose to define the reduction vectors as being homogeneous over the different groups, that is,


(9) $$a_{\nu i}=\left\{ \begin{array}{cc} 1/m_{\nu } & \text{if }i\in G_{\nu },\\ 0 & \text{otherwise}. \end{array}\right.$$

The last equation defines the *homogeneous reduction*. The justification behind this choice is twofold. First, in the absence of any additional constraint, all nodes in a group should contribute equally to the corresponding observable. Second, in perfectly homogeneous modular networks the homogeneous observables defined by Eq. 9 are the only ones that exactly solve the compatibility equations and make this reduction coincide with the spectral reduction defined below (see Proposition 2 in the Supplementary material).

#### Spectral reduction

In what we call the *spectral reduction*, we go one step further and approximate *f* and *g* by first-order Taylor polynomials around the appropriate observables:


(10a) $$\begin{array}{r} \;f(x_{i})\approx f(X_{\nu })+f^{\prime }(X_{\nu })(x_{i}-X_{\nu }), \end{array}$$

(10b) $$\begin{array}{rl} g(x_{i},x_{j}) & \approx g(X_{\nu },X_{\rho })+g_{1}(X_{\nu },X_{\rho })(x_{i}-X_{\nu })\\ & \;+g_{2}(X_{\nu },X_{\rho })(x_{j}-X_{\rho }), \end{array}$$

where $i\in G_{\nu },j\in G_{\rho }$, and $g_{1},g_{2}$ denote the partial derivatives of *g* with respect to its first and second arguments, respectively. Note that if *f* and *g* are affine on their arguments, then this approximation is in fact *exact*. Substituting these expressions into Eq. 6 does not, however, yield a closed dynamics. As stated in Proposition 1 (see section “Approximate reduced dynamics” of the Supplementary material), the reduction vectors must fulfill the following conditions to close the system:


(11a) $$K_{\nu \rho }{\hat{a}}_{\nu }=\mu_{\nu \rho }{\hat{a}}_{\nu },$$

(11b) $$W_{\nu \rho }^{T}{\hat{a}}_{\nu }=\lambda_{\nu \rho }{\hat{a}}_{\rho },$$

for ν,ρ∈{1,…,n}. The vector ${\hat{a}}_{\nu }\in R^{m_{\nu }}$ is defined by the components of $a_{\nu }$ that correspond to nodes within group $G_{\nu }$ (the other elements are 0 by definition). We call ${\hat{a}}_{1},\ldots ,{\hat{a}}_{n}$ the *partial reduction vectors*. The matrices $\mu =(\mu_{\nu \rho }{)}_{\nu ,\rho }$ and $\lambda =(\lambda_{\nu \rho }{)}_{\nu ,\rho }$, both of dimension n×n, are sets of parameters to be determined. We note that Eqs. 11a and 11b need to be imposed whenever the *g* function varies with its first and its second arguments, respectively. This means, for example, that reducing a system in which g(x,y)=g(y) does not require Eq. 11a to be fulfilled.

As in Ref. (27), we refer to conditions 11a and 11b as the *compatibility equations*. They relate the reduction vectors with the adjacency and weighted in-degree matrices, independently of the functions that define the node dynamics. It is therefore solely the structure of interactions in the network that shapes and constrains the construction of the observables in our approach.

As detailed in the proof of Proposition 1 of the Supplementary material (section “Approximate reduced dynamics”), once the compatibility equations are fulfilled, the parameters $\lambda_{\nu \rho }$ and $\mu_{\nu \rho }$ coincide with the group-to-group averaged in-degree defined by Eq. 8,


(12a) $$\mu_{\nu \rho }=W_{\nu \rho }$$

(12b) $$\lambda_{\nu \rho }=W_{\nu \rho },$$

and the approximate temporal evolution of the νth observable is given by


(13) $${\dot{X}}_{\nu }\approx f(X_{\nu })+\sum_{\rho =1}^{n}W_{\nu \rho }g(X_{\nu },X_{\rho }).$$

As in the homogeneous reduction, this dynamics preserves the form of the original dynamics and includes a n×n*reduced adjacency matrix*$W=(W_{\nu \rho }{)}_{\nu ,\rho }$ between the different observables, defined by Eq. 8.

Thus, in the spectral reduction, mapping the original dynamics into the reduced dynamics requires (a) solving the compatibility equations to find the reduction vectors $a_{1},\ldots ,a_{n}$, and (b) computing the reduced adjacency matrix $(W_{\nu \rho }{)}_{\nu ,\rho }$ from the reduction vectors. The second step is straightforward but not the first one. Solving the compatibility equations can be problematic because, except in very particular cases, these equations cannot be fulfilled simultaneously. In the next section, we propose a method for finding an approximate solution, which takes into account the spectral properties of the group-to-group weighted in-degree and adjacency matrices. This is why we call it the *spectral reduction*. Fig. 1 shows a schematics of the spectral reduction process.

### Solving the compatibility equations

The spectral reduction requires solving the compatibility equations 11a and 11b to determine the reduction vectors. Eq. 11a is an eigenvalue–eigenvector equation for the partial reduction vector ${\hat{a}}_{\nu }$. Eq. 11b is more involved because it includes a crossed dependency between different partial reduction vectors. Corollary 2 in the Supplementary material (section “Equivalent forms for the compatibility equations when the adjacency matrix is positive”) provides a strategy to transform Eqs. 11a and 11b into an equivalent set of equations which are easier to solve. This can be done when the original adjacency matrix is positive and it can be summarized as follows. If we assume the partial reduction vectors ${\hat{a}}_{1},\ldots ,{\hat{a}}_{n}$ to be also strictly positive, Eqs. 11a and 11b are equivalent to the following *decoupled* (i.e. without crossed dependencies) compatibility equations


(14a) $$K_{\nu \rho }{\hat{a}}_{\nu }=\mu_{\nu \rho }{\hat{a}}_{\nu },$$

(14b) $${W^{\prime }}_{\nu \rho }{\hat{a}}_{\nu }=\lambda_{\nu \rho }^{\prime }{\hat{a}}_{\nu }$$

for ν,ρ∈{1,…,n}, where


(15a) λνρ′:={λννif ν=ρ,λνρλρνmif ν≠ρ,

(15b) $$\begin{array}{rl} {W^{\prime }}_{\nu \rho } & \;:\;=\left\{ \begin{array}{cc} W_{\nu \nu }^{T} & \text{if }\nu =\rho ,\\ W_{\rho \nu }^{T}W_{\nu \rho }^{T} & \text{if }\nu \neq \rho . \end{array}\right. \end{array}$$

The decoupled form 14b for fixed ν and variable ρ is easier to treat because it consists of a set of *n* equations on ${\hat{a}}_{\nu }$ and $\{\lambda_{\nu \rho }^{\prime }{\}}_{\rho }$ that we can try to solve independently for each ν. These equations state that ${\hat{a}}_{\nu }$ has to be, simultaneously, the dominant eigenvector of a collection of *n* matrices (the dominant condition being a consequence of the Perron-Frobenius Theorem). Eq. 14a states that ${\hat{a}}_{\nu }$ should also be an eigenvector of the *n* diagonal matrices $K_{\nu 1},\ldots ,K_{\nu n}$.

Let us first address the problem of solving Eq. 14b for a fixed ν and with ρ ranging in {1,…,n}. In general, the equations in this set are not simultaneously solvable because, except in very particular cases, the matrices involved do not share the dominant eigenspace. To find an approximate solution, we first assume that the scalars $\{\lambda_{\nu \rho }^{\prime }{\}}_{\rho }$ are the dominant eigenvalues of the matrices involved (this would be the case if the equations could be solved exactly). Then, we find a vector ${\hat{a}}_{\nu }$ that has sum 1 and minimizes the sum of the quadratic errors associated to Eqs. 14b, see section “An approximate solution to the compatibility equations that involve the adjacency matrix” and Fig. S1 in the Supplementary material for details.

We have addressed the problem of solving Eq. 11b but not Eq. 11a. Assuming again that W is a positive matrix and that we want the reduction vectors to be positive, Eqs. 11a and 11b are equivalent to Eqs. 14a and 14b; for a fixed ν, these are eigenvector–eigenvalue equations for the vector ${\hat{a}}_{\nu }$. Thus, it could be tempting to try to find an approximate solution following the strategy proposed above just by adding Eqs. 14a for all ρ to the list of eigenvector–eigenvalue equations that involve ${\hat{a}}_{\nu }$. However, contrary to what happens with Eqs. 14b, we do not have a criterion to choose the corresponding scalars $\mu_{\nu \rho }$. In principle, any of the diagonal entries of matrix $K_{\nu \rho }$ could be chosen as $\mu_{\nu \rho }$, but the resulting error and solution vector ${\hat{a}}_{\nu }$ could be very different depending on this choice.

To solve this issue, we apply the following procedure: for a fixed ν, we first approximately solve Eqs. 14b to determine ${\hat{a}}_{\nu }$. Once this vector is specified, for every ρ we compute the scalar $\mu_{\nu \rho }$ that minimizes the quadratic error associated to Eq. 14a, which is given by


(16) $$\mu_{\nu \rho }=\frac{{\hat{a}}_{\nu }^{T}K_{\nu \rho }{\hat{a}}_{\nu }}{\|{\hat{a}}_{\nu }{\|}^{2}}$$

(see Lemma 3 in the Supplementary material). In doing so, we no longer assume that Eq. 12a holds, and this leads to a reduced dynamics that incorporates a correction factor which depends on matrix $\mu =(\mu_{\nu \rho }{)}_{\nu ,\rho }$:


(17) $$\begin{array}{rl} {\dot{X}}_{\nu } & \approx f(X_{\nu })+\sum_{\rho =1}^{n}W_{\nu \rho }\;g(X_{\nu },X_{\rho })\\ & \;+\sum_{\rho =1}^{n}(\mu_{\nu \rho }-W_{\nu \rho })g_{1}(X_{\nu },X_{\rho })X_{\nu } \end{array}$$

(see section “Correction when the compatibility equations cannot be solved exactly” of the Supplementary material for details).

Notice that the reduced dynamics described by Eq. 17 reduces to the one presented earlier in Eq. 13 when the compatibility equations that involve the weighted in-degree matrix are solved exactly or when the *g* function does not depend on its first argument. Also, when the node partition is such that the connectivity properties of nodes in the same group are very similar, we can expect each matrix $K_{\nu \rho }$ to approximately be a multiple of the identity matrix, and in this case conditions [14a] and [12a] are automatically fulfilled for any ${\hat{a}}_{\nu }$. This suggests that the reduced dynamics [13] is quite accurate when the groups are composed of nodes of similar connectivity properties, which is one of the main assumptions behind our reduction methods.

A summary of the homogeneous and the spectral dimension-reduction methods is given in Table 1.

**Table 1.** Summary of the homogeneous and the spectral dimension-reduction methods.

Original adjacency matrix	$W=(w_{ij}{)}_{i,j=1}^{N}$
Original dynamics	${\dot{x}}_{i}=f(x_{i})+\sum_{\;j=1}^{N}w_{ij}g(x_{i},x_{j})$
Groups	$G_{1},\ldots ,G_{n}$ , $G_{\nu }\cap G_{\rho }=\emptyset \text{ if }\nu \neq \rho$, $\overset{n}{\underset{\nu =1}{⋃}}G_{\nu }=\{1,\ldots ,N\}$, $m_{\nu }\;:\;=|G_{\nu }|$
Reduction vectors	$a_{\nu }=(a_{\nu i}{)}_{i=1}^{N}$ , $a_{\nu i}=0\text{ if }i\notin G_{\nu }$, $\sum_{i=1}^{N}a_{\nu i}=1$
Partial reduction vectors	${\hat{a}}_{\nu }=({\hat{a}}_{\nu k}{)}_{k=1}^{m_{\nu }}=(a_{\nu i}{)}_{i\in G_{\nu }}$ , $\sum_{k=1}^{m_{\nu }}{\hat{a}}_{\nu k}=1$
Reduced adjacency matrix	$W=(W_{\nu \rho }{)}_{\nu ,\rho =1}^{n}$ , $W_{\nu \rho }\;:\;=\sum_{i\in G_{\nu },j\in G_{\rho }}a_{\nu i}w_{ij}$
Observables	$X_{\nu }=\sum_{i=1}^{N}a_{\nu i}x_{i}=\sum_{k=1}^{m_{\nu }}{\hat{a}}_{\nu k}{\hat{x}}_{\nu k}$ , ${\hat{x}}_{\nu }=({\hat{x}}_{\nu k}{)}_{k=1}^{m_{\nu }}=(x_{i}{)}_{i\in G_{\nu }}$

Reduction	Conditions	Reduced dynamics
Homogeneous	${\hat{a}}_{\nu }=\frac{1}{m_{\nu }}(1,\ldots ,1{)}^{T}$	${\dot{X}}_{\nu }\approx f(X_{\nu })+\sum_{\rho =1}^{n}W_{\nu \rho }g(X_{\nu },X_{\rho })$
Spectral	$K_{\nu \rho }{\hat{a}}_{\nu }=\mu_{\nu \rho }{\hat{a}}_{\nu }\;\text{(C1)}$	If Eqs. (C1), (C2) can be solved exactly,
	$W_{\nu \rho }^{T}{\hat{a}}_{\nu }=\lambda_{\nu \rho }{\hat{a}}_{\rho }\;\text{(C2)}$	${\dot{X}}_{\nu }\approx f(X_{\nu })+\sum_{\rho =1}^{n}W_{\nu \rho }g(X_{\nu },X_{\rho })$
	(compatibility equations)	Otherwise, approximately solve Eq. (C2), set $\mu_{\nu \rho }=\frac{{\hat{a}}_{\nu }^{T}K_{\nu \rho }{\hat{a}}_{\nu }}{\|{\hat{a}}_{\nu }{\|}^{2}}$ and ${\dot{X}}_{\nu }\approx f(X_{\nu })+\sum_{\rho =1}^{n}W_{\nu \rho }g(X_{\nu },X_{\rho })+\sum_{\rho =1}^{n}(\mu_{\nu \rho }-W_{\nu \rho })g_{1}(X_{\nu },X_{\rho })X_{\nu }$

To solve the compatibility equations (exactly or approximately if an exact solution does not exist) for positive adjacency matrices, see the procedure defined in section “Solving the compatibility equations.”

## Exploring the spectral reduction

So far, we have presented a method for reducing a given *N*-dimensional dynamical system on a network into an *n*-dimensional one whose variables, the observables, represent weighted averages of the node activities of the original network. We assumed that the nodes in the original network are organized into *n* groups of similar connectivity properties. In the reduction, the observables are constructed so that each of them represents the activity within each of these groups. We have also presented a homogeneous reduction in which the observables are simply the homogeneous averages of node activities within each group and where the connection weight from group ρ to group ν in the reduced system is given by the mean weighted in-degree of nodes in group ν considering only connections from group ρ. The homogeneous reduction is then the simplest strategy to coarse-grain the original system and it has been introduced as a baseline for comparison with the spectral reduction.

The accuracy of the reduction is expected to strongly depend on the number of groups *n* and on the precise arrangement of the nodes into the different groups. Defining the groups and anticipating what *n* should be to get a proper reduction is, however, a difficult task and there is no clear method to that end. A large repertoire of community detection algorithms on networks has been developed in the last years, which include spectral-based methods, algorithms that use information theory analysis, and Bayesian inference methods that fit the input network to modular graph models (41–43), to cite only some examples. The issue of detecting such groups, despite being necessary for our reductions to be accurate, is not central to the present work. When studying the dimension-reduction methods that we have described earlier we will assume that the networks have been previously analyzed and the main communities have been already detected. We will nonetheless propose ways to refine a given node partition to obtain a richer and more accurate reduced system when the node heterogeneity within the given groups is too large. This will be a fruitful strategy when dealing with networks whose connectivity is highly heterogeneous.

We expect a proper reduction to be able to capture some salient features of the original dynamical system. In a system at equilibrium, such a feature can be the presence of bifurcations, also called critical transitions, which are qualitative changes of the system’s state in response to perturbations of some structural or dynamical parameters (13, 14). The detection of the tipping points, i.e. parameter values at which bifurcations occur, has proved to be crucial for anticipating dynamical regime shifts in a large variety of real-world complex systems, be them climatic (44), neurological (45), economic (46), or ecological (47). We would like the reduced dynamics to capture these critical parameters even if the precise activity at equilibrium is not necessarily obtained with high accuracy.

In what follows, we study the ability of the spectral reduction to predict bifurcation diagrams, including their tipping points, of different dynamical systems as we vary the overall strength of all the interactions in the network. We compare the results to those obtained from the homogeneous reduction. As our method for solving the compatibility equations requires the adjacency matrix to be positive, in this work we restrict ourselves to positive matrices. We can easily transform a nonnegative weighted or binary adjacency matrix into a positive one by simply assuming that the missing interactions are arbitrarily weak.

### Node dynamics

The proposed method for dimension reduction can be applied to a node dynamics defined by Eq. 1 for arbitrary functions *f* and *g* of class $C^{1}$. In this paper, we have chosen *f* and *g* so as to model three types of dynamics on networks: a form of neuronal dynamics, a model of infectious disease spreading (susceptible-infected-susceptible, SIS, dynamics) and an ecological dynamics, see section “Examples of node dynamics” of the Supplementary material.

### Exact versus reduced bifurcation diagrams for networks with block structure

We first assess the extent to which the homogeneous and spectral reduction methods are able to reflect the system’s sensitivity to parameter changes in random networks with known block structure. For this, we homogeneously vary the magnitude of all the interactions so as to force the system to transition between bistable regimes and regimes characterized by a single equilibrium point. By modifying the interaction strengths back and forth and integrating the system towards equilibrium, we can capture a bifurcation diagram that reflects these transitions (see section “Computing the bifurcation diagrams” of the Supplementary material for further details).

To compare the reduced and the exact bifurcation diagrams, we plot a weighted average of the *n* observables at equilibrium for the exact and reduced systems, ⟨X⟩, as a function of the average weighted in-degree of the reduced system, ⟨K⟩. The magnitude ⟨X⟩ is the observables’ average weighted by group size and reflects the overall state of the system:


(18) $$\langle X\rangle \;:\;=\frac{1}{N}\sum_{\nu =1}^{n}m_{\nu }X_{\nu }.$$

The parameter ⟨K⟩ is defined by


(19) $$\langle K\rangle \;:\;=\frac{1}{N}\sum_{\nu =1}^{n}m_{\nu }K_{\nu },\;K_{\nu }\;:\;=\sum_{\rho =1}^{n}W_{\nu \rho }.$$

#### Homogeneous networks

We start by considering random networks constructed according to the directed version of the stochastic block model (SBM) (48), in which nodes are arranged into *n* modules and binary connections appear independently with probabilities that depend on the node membership. By varying these probabilities, we can create a full range of network structures, from assortative ones, in which nodes are densely connected to nodes in their same module, to dissortative networks, in which interactions within nodes in the same module are rare.

**Fig. 2.**
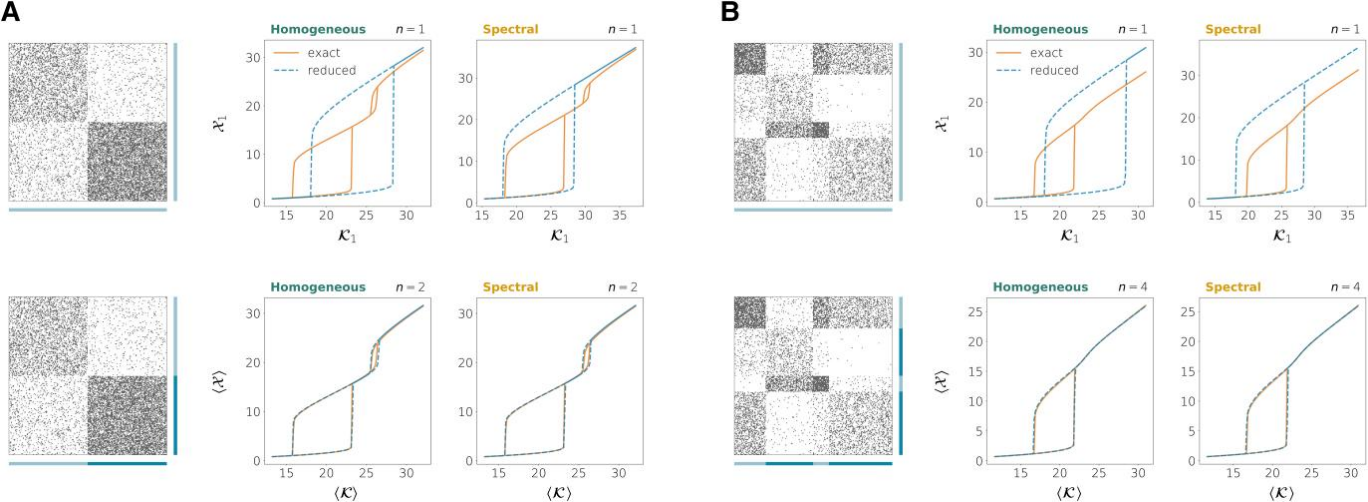
Exact versus reduced bifurcation diagrams for homogeneous directed networks generated from the SBM and neuronal dynamics (Eq. S88 with τ=0.3, μ=10). The left panels show the adjacency matrices and the bars on their right and bottom sides indicate the node partition used for the reductions. When the dimension of the reduction is n>1, the bifurcation diagrams show the value of the average observable at equilibrium defined by Eq. 18. The parameter on the *x*-axis is the average weighted in-degree of the reduced system defined by Eq. 19. A) Network on N=200 nodes and two communities of the same size in which the mean connection densities are $p_{11}=0.3$, $p_{12}=0.05$, $p_{21}=0.1$, $p_{22}=0.6$. B) Network on N=200 nodes and four communities with relative sizes 0.2, 0.3, 0.1, 0.4 and mean connection densities $p_{11}=0.75$, $p_{12}=0.05$, $p_{13}=0.6$, $p_{14}=0.3$, $p_{21}=0.1$, $p_{22}=0.2$, $p_{23}=0.03$, $p_{24}=0.002$, $p_{31}=0.01$, $p_{32}=0.5$, $p_{33}=0.9$, $p_{34}=0.05$, $p_{41}=0.35$, $p_{42}=0.03$, $p_{43}=0.1$, $p_{44}=0.25$.

**Fig. 3.**
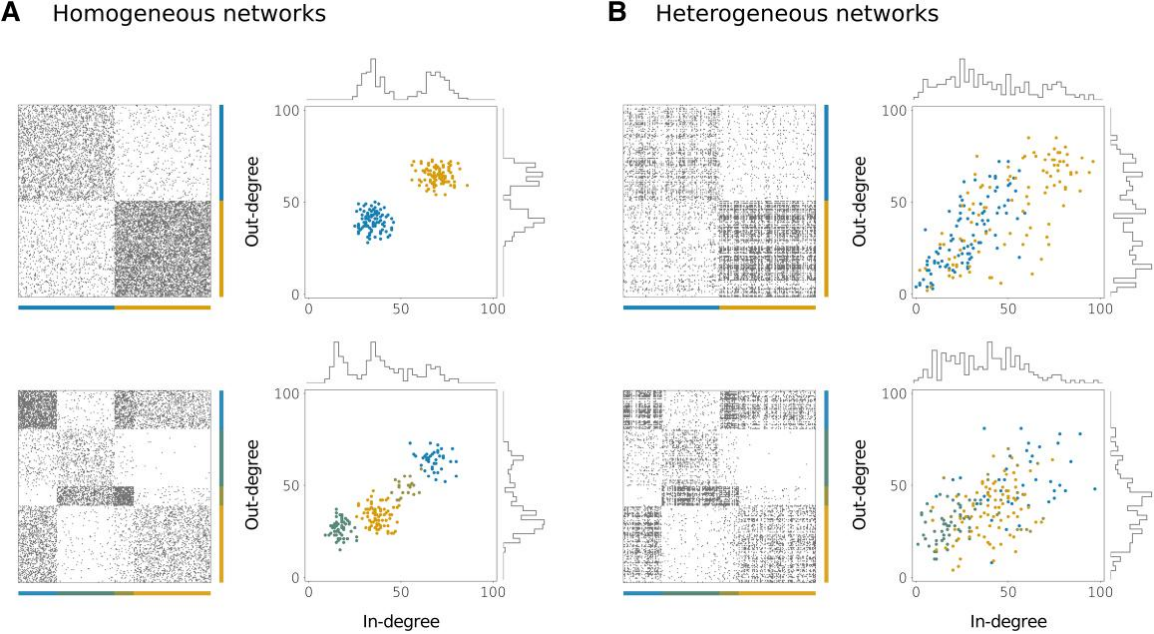
Adjacency matrices and in/out-degrees for homogeneous A) and heterogeneous B) directed networks. Each color corresponds to one community. The networks are analogous to those of Figs. 2 and 4.

Fig. 2 compares the two reductions when we take the whole network as a single group (n=1) and when the node partition is that of the true communities in the network. When the number of communities is larger than 1, neither the homogeneous nor the spectral reductions are able to reproduce the correct bifurcation diagram with n=1 (although the spectral reduction provides a better approximation of the bifurcation points) but both of them yield very accurate results when the true communities are provided and *n* is increased accordingly. The two methods exhibit a very similar performance in this case. The reason is that the SBM (in the dense regime and when the network’s size is large) tends to generate quite homogeneous networks, in which there is small variability in terms of connectivity among the nodes that are in the same group (see Fig. 3A). In fact, in any modular network with *perfectly* homogeneous connectivity (that is, when the connection strength $w_{ij}$ only depends on the membership of nodes *i* and *j*, for all i,j), the homogeneous and the spectral reductions coincide as long as the true communities are identified (see Proposition 2 in the Supplementary material). In general, this makes the homogeneous reduction enough for predicting the bifurcation diagram in *nearly* homogeneous networks, although there are situations in which correctly identifying the communities might not guarantee an accurate prediction of the bifurcation points by the reduced dynamics. We will come back to this issue later.

#### Heterogeneous networks

**Fig. 4.**
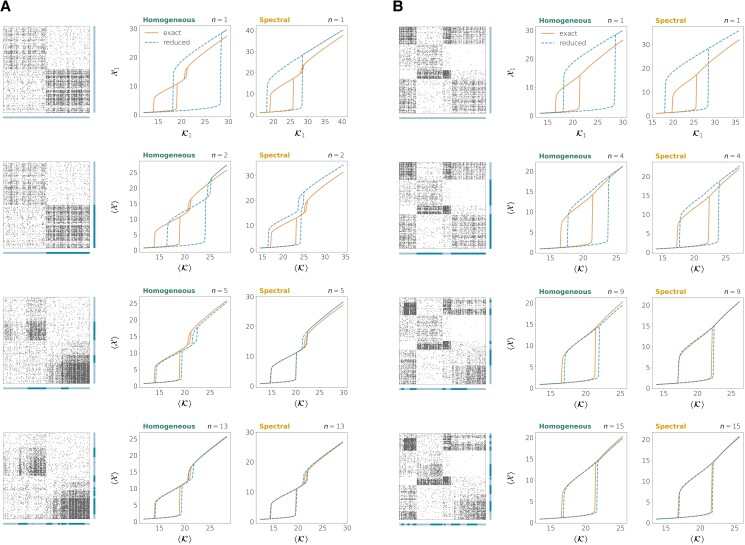
Exact versus reduced bifurcation diagrams for heterogeneous directed networks and neuronal dynamics (Eq. S88 with τ=0.3, μ=10). A) Network on N=200 nodes and two communities with the same sizes and same mean connection densities as in Fig. 2A. B) Network on N=200 nodes and four communities with the same sizes and same mean connection densities as in Fig. 2B.

We now analyze the performance of the two methods when the network has a known modular structure and there is also a large heterogeneity in the connectivity properties of nodes that are in the same module. Inspired by the Chung–Lu model (49, 50), we created a modified version of the directed SBM that can incorporate an important variability of in- and out-degrees within nodes belonging to the same community (see section “Construction of heterogeneous networks with communities” of the Supplementary material as well as Fig. 3B). In this case, the homogeneous and the spectral methods show different performances, even when the true community structure is used to define the groups in the reductions. The spectral reduction tends to provide more accurate bifurcation diagrams. Yet, when the dimension *n* is lower or equal to the number of communities, even the spectral method might yield results that are not quantitatively accurate for some dynamics. This is illustrated by the two upper rows of Fig. 4, that show the performance of our reduction methods on heterogeneous networks with the same dynamics, the same number of communities and the same mean connection densities as in the homogeneous networks of Fig. 2.

This lack of accuracy is caused by the large heterogeneity among the nodes in the same group. In a way, the number of *effective* groups in these networks is larger than the number of communities used to construct the network. Therefore, more refined partitions should be defined in order for the reduced dynamics to accurately predict the bifurcation diagrams.

To refine a given partition, we used a procedure whose goal is to divide the groups into subgroups so that the variability within nodes that are in the same subgroup is reduced. This variability could correspond to different connectivity attributes of nodes. As an example, we focused on the weighted in/out-degrees from/to the other groups (see section “Partition refinement” of the Supplementary material). This allows us to create nested partitions from an original partition while progressively increasing the number of groups *n* and, with it, the dimension of the reduced dynamics.

**Fig. 5.**
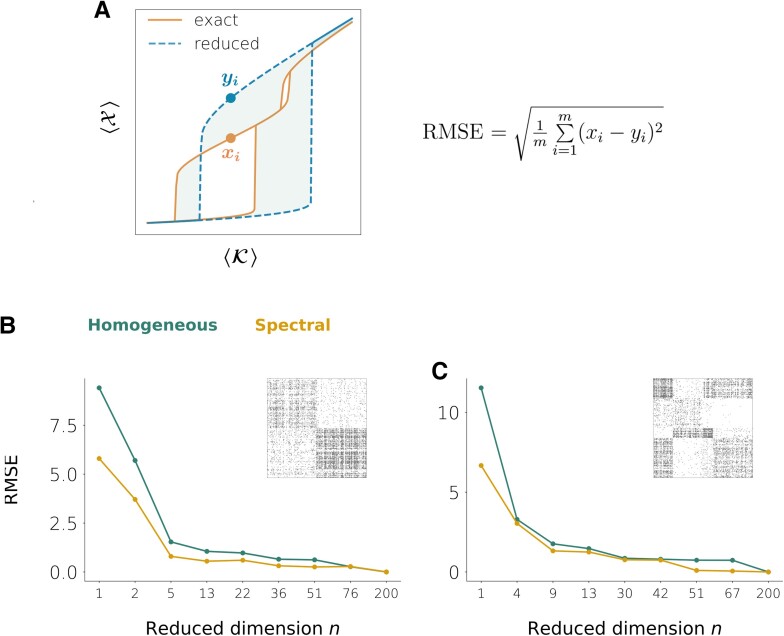
Reduction error as the reduced dimension *n* is increased. A) Root-mean-square-error (RMSE) between the exact and the reduced bifurcation diagrams as a measure of the reduction error. B) Network on N=200 nodes and two communities with the same mean connection densities and same dynamics as in Fig. 4A. C) Network on N=200 nodes and four communities with the same mean connection densities and same dynamics as in Fig. 4B.

As shown in the lower rows of Fig. 4, the partition refinements improve the quality of the reductions, especially for the spectral method. We can measure the reduction’s quality by the root-mean-square-error (RMSE) between the true bifurcation diagram and the one obtained from simulating the reduced dynamics (Fig. 5A). Fig. 5B and C shows the RMSE as we increase the reduced dimension *n*.

Our results suggest that the spectral reduction is able to cope better with heterogeneities in the adjacency matrix. The comparison between these networks and their homogeneous counterparts of Fig. 2 indicates that the effective dimension of the heterogeneous networks is larger, although it is still smaller compared to that of the original dynamics (N=200). We observe a similar trend in other network examples provided with different node dynamics (Fig. S2).

#### Relevant heterogeneities in homogeneous networks

**Fig. 6.**
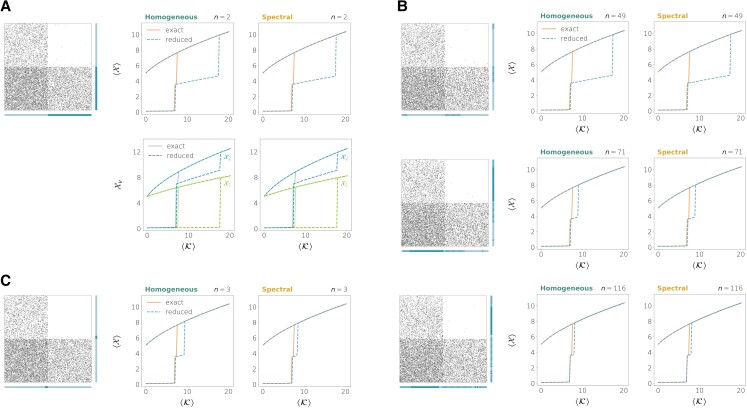
Exact versus reduced bifurcation diagrams for a directed network on N=200 nodes generated from the SBM with two communities and ecological dynamics (Eq. S91 with B=0.1, C=1, K=5, D=6, E=0.9, H=0.1). The network has N=200 nodes and two communities of the same size and with mean connection densities $p_{11}=0.2$, $p_{12}=0.001$, $p_{21}=0.5$, $p_{22}=0.3$. A) Bifurcation diagrams when the true communities are provided (n=2). The bottom panels show the exact versus reduced individual observables at equilibrium. B) Bifurcation diagrams obtained as the original partition is progressively refined so as to reduce the intra-group weighted degree variability (n=49, n=71, n=116). C) Bifurcation diagrams when an ad hoc partition is defined in which the nodes in group 1 that receive input from group 2 constitute a third group (n=3).

Even in homogeneous networks, there are situations in which correctly identifying the communities might not guarantee an accurate prediction of the bifurcation points by the reduced dynamics. Fig. 6 shows the bifurcation diagram corresponding to an ecological dynamics on a network composed of two communities. The connection density is relatively high within each group and also from group 1 to group 2 but not the other way around: there exist few connections from group 2 to group 1. When the initial state of the system is that of a low species abundance in both groups, the system stays in that state until the overall connection strength reaches a critical threshold that makes group 2 (the one with denser connectivity) jump to a high abundance state (Fig. 6A, blue continuous curves in the bottom plots). This first jump is well captured by the reduced dynamics (blue dashed curves). The reduced systems also predict that group 1 should remain in the low abundance state until a second threshold in connection strength is reached (green dashed curves). This threshold is nevertheless not well predicted because group 1 shifts to a high abundance state much earlier in the complete system (green continuous curves). The discrepancy between the exact and the reduced bifurcation diagrams is large for both reduction methods.

The source of the discrepancy is the heterogeneity in the input that nodes in group 1 receive from group 2: the majority of nodes in group 1 do not receive any input from group 2 but a few of them get input from exactly one node in group 2. In the original network, the input that this last set of nodes receives is enough for them to jump to a high abundance state right after the jump of group 2. Due to the high density within group 1, they recruit the other nodes in their group and the result is that the whole group shifts to a high abundance state right after group 2 does so. The reduced system with 2 observables (either homogeneous or spectral), however, only takes into account the *average* input from group 2 to group 1. This average is not large enough to drive group 1 to the high abundance state unless the connection strengths are much larger.

This suggests that the *deviations* from the average input are the cause of the mismatch between the original and the reduced system. In this example, a small deviation in the input received can change the whole state of the system, whereas the reduced system is not able to capture it because it relies on group input averages.

The problem can be solved by refining the partition until the inter-group input variability is small enough. We applied the same partition refinement described in the previous section and we found that a partition into a large number of groups (n≈116 for a network on N=200 nodes) is needed to properly capture the second transition (Fig. 6B). This is so because our refinement method is designed to reduce the difference between the maximal and minimal weighted degrees in a given group, regardless of the magnitude of these degrees (i.e. a degree difference of 1 is treated the same way when the degrees are of the order of 20 and when they are of the order of 1). The result is that a very fine refinement is needed to separate the nodes in group 1 that cause the whole group to jump.

But it is possible to define an ad hoc partition that makes the second transition be well captured by the reduced system. If we separate from the rest the nodes in group 1 that have in-degree of 1 from group 2, we get a 3-group partition for which the reduced dynamics exhibits a reasonably good accuracy (Fig. 6C), similar to that obtained for the refinement with n=116. This illustrates that, in some systems, small heterogeneities in connectivity can make the reduced system fail in predicting the true transitions but this might be solved by properly choosing the node partition.

### Robustness with respect to partition choice

Until now we have analyzed the performance of the homogeneous and the spectral reductions for “good” partitions, that is, partitions that group nodes with similar connectivity properties. In particular, we have used partitions that correspond to the true modular organization of the network, together with successive refinements based on weighted in/out-degree variability. Doing so is necessary for the reductions to provide accurate results. However, in many situations we will not have access to any information regarding the presence of communities in the network. Instead, we will have to infer this information by analyzing the network structure, a task that can be problematic and might result in far from optimal partitions. Thus, a relevant question to be addressed is to what extent our reduction methods are sensitive to the partition choice.

**Fig. 7.**
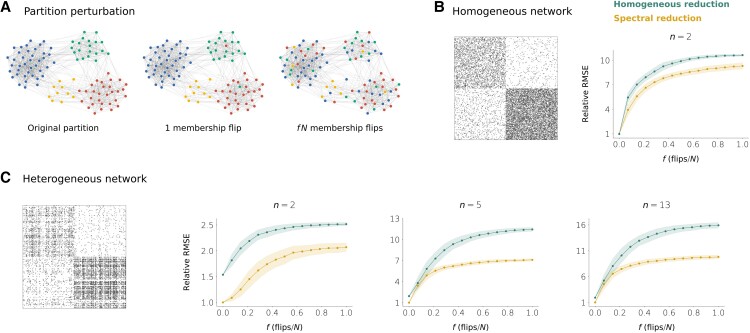
Sensitivity of the homogeneous and spectral reductions to partition perturbation. A) Schematics of the partition perturbation procedure. B, C) RMSE (relative to the RMSE of the spectral reduction when f=0) as a function of *f* for the homogeneous and the spectral reductions. For each *f*, 300 random perturbations of the original partition were created. The lines show the average relative RMSE ± the standard deviation of the ensemble. B) Results for the homogeneous network of Fig. 2A when the original partition is the one used in that figure (n=2). C) Results for the heterogeneous network of Fig. 4A. The original partitions are the ones shown in Fig. 4A (n=2, n=5, n=13).

We analyzed the performance of our reduction methods as we randomly perturb a nearly optimal partition. Given an original partition $P_{0}$, we select a pair of nodes at random and we flip their group membership. For f∈[0,1], we repeat this process ⌊fN⌋ times to get a new partition $P_{f}$ that preserves the number of groups and the group sizes of $P_{0}$ but which otherwise is a randomized version of $P_{0}$ (Fig. 7A). We can repeat this many times to get an ensemble of perturbations of $P_{0}$ for a given *f* and then plot the average and the standard deviation of the discrepancy obtained according to each reduction method. Again, we define the discrepancy as the RMSE between the true bifurcation diagram and the one obtained from simulating the reduced dynamics (Fig. 5A).

Fig. 7B and C shows the results for the two-community networks (homogeneous and heterogeneous) explored in Figs. 2A and 4A. We took as the original partitions the ones used in these figures for n=2 (homogeneous network) and n=2, n=5, n=13 (heterogeneous network). The plots show the average RMSE (± standard deviation) relative to that of the spectral reduction for the original partition (f=0) as a function of *f*. Several conclusions can be derived from the results. First, the original partitions are close to be optimal because perturbing them results in larger errors on average. Second, the spectral method performs better than the homogeneous method, regardless of *f*. Third, the difference in performance between the two reduction methods is smaller for the homogeneous network, so the spectral method might be especially useful when dealing with heterogeneous networks. However, in the homogeneous network it is still preferable to use the spectral method, except if the true communities are perfectly identified (f=0, case in which the two methods exhibit the same performance). Finally, except in the heterogeneous network when n=2, the average error increase with respect to the case f=0 tends to be smaller for the spectral reduction, which suggests that this reduction is more robust to ill-posed node partitions.

### Dimension reduction on real networks

**Fig. 8.**
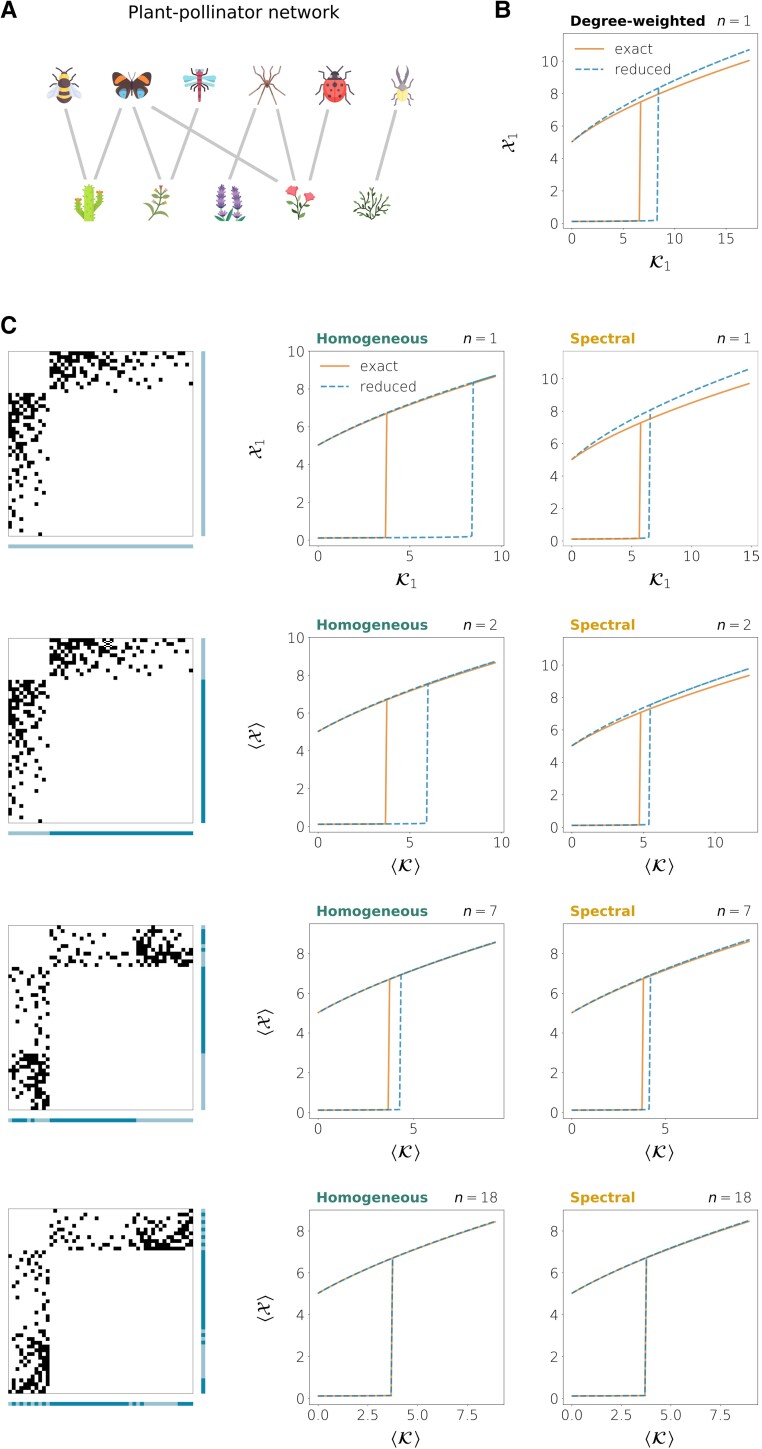
Ecological dynamics (Eq. S91, same parameters as in Fig. 6) on a plant–pollinator network of insects and plants in a Canary Island, Spain (51). The interaction graph is bipartite, binary, and undirected and contains 11 plants and 38 pollinators (N=49). A) Schematics of a plant–pollinator network. Icons have been taken from Flaticon.com. B) Bifurcation diagram obtained from the degree-based reduction defined in Ref. (20). C) Bifurcation diagrams for the homogeneous and the spectral methods when the whole network is taken as a single group (n=1) and for successive refinements of a partition on n=2 groups, each one representing one species type (plant or pollinator).

We finally explore the performance of the homogeneous and spectral reduction methods when applied to dynamical systems on three networks obtained from real data. We first consider the mutualistic ecological dynamics given by Eq. S91 together with a plant–pollinator network from the sub-alpine desert of Tenerife, in the Canary Islands (51). It consists of 11 flowering plant species and 38 pollinator species (2 bird and 36 insect species), N=49. The authors assumed that an interaction exists between a plant and a pollinator whenever the pollinator had been observed probing for nectar or eating/collecting pollen from the plant. The resulting network of interactions is undirected and bipartite, as the imaginary plant–pollinator network depicted in Fig. 8A. When the interaction strength is large enough, the system has a single stable equilibrium state with large species abundances. But as the interactions are weakened, there is a transition to a bistable regime in which a state characterized by very low species abundances is also possible. In the example shown here, this low abundance state is not an extinction state because we chose a positive migration rate *B*, meaning that even if the species went extinct, migration from other territories would make their numbers grow again. In any case, the presence of such a low abundance state indicates that if the species’ numbers are not large enough, the entire ecosystem can collapse into a state in which species can no longer benefit from the interaction with others and can only be maintained by migration. This kind of collapse might have catastrophic consequences for the ecosystem. The transition point is captured quite well by the spectral reduction, even when the reduction is one-dimensional (Fig. 8C). The spectral method for n=1 also outperforms the degree-based one-dimensional reduction defined by Gao et al. (20) (Fig. 8B). As nodes are classified into groups of similar connectivity (by separating plants from pollinators first, n=2, and then by refining this partition), the tipping point is better approximated, the improvement being more evident in the homogeneous reduction, which provides poor results when *n* is not large enough.

The second example is an undirected and binary social network based on Facebook contacts (52) where N=362. To classify the nodes into groups of similar connectivity properties, we used the minimize_blockmodel_dl algorithm from the graph-tool Python library, which fits a given network into an SBM (53). The algorithm detected 15 communities and this partition was then refined so as to reduce the degree variability within each community. We simulated the spread of an epidemics in the network, given by the SIS dynamics of Eq. S90. An important feature to study in such a system is whether the stable equilibrium is disease-free, which means that the disease will disappear completely in the long term, or if it is endemic, in which case the disease will remain. These two types of equilibria are characterized by ⟨X⟩=0 and ⟨X⟩>0, respectively. While the interactions are weak, the pathogen does not propagate enough through the network and the stable equilibrium is disease-free. The point at which an endemic stable equilibrium appears is almost perfectly captured by the one-dimensional spectral reduction (Fig. S3C), which again outperforms the degree-based reduction introduced by Gao et al. (20) (Fig. S3B). The homogeneous reduction is unable to capture this critical point even at n=19.

These two applications of the spectral reduction method to dynamics on real networks highlight the power of this dimension-reduction strategy as a way to understand the behavior of complex systems by means of simpler systems: in both cases, a reduction of order 1 (whose equilibria could be found by hand) was enough to provide a faithful picture of what the true bifurcation diagram looks like.

In the third example, we studied a neuronal dynamics on the *Caenorhabditis elegans* connectome described in Ref. (54) where N=279. Nodes represent single neurons and the weighted and directed interactions represent connections among them (Fig. S4A). We partitioned the nodes as we did for the contact network. The bifurcation diagram in this case is complex, with multiple bifurcation events, and it is not well represented by neither the degree-based reduction nor our reduced systems when the dimension is too small (Fig. S4B and C). This can be interpreted as a signature of the system having a large effective dimension, much larger than the other two example systems. In general, for a fixed reduced dimension *n*, the spectral reduction provides a much more accurate picture of the bifurcation diagram than the homogeneous one.

## Discussion

We have presented a strategy to reduce the dimension of a dynamical system on a network of interactions. The variables of the reduced system, the observables, are weighted averages of the activities within *n* groups of nodes in the network. The node partition is defined a priori based on the structure of the adjacency matrix and it is supposed to maximize the similarity of nodes that are in the same group. The key step in our reduction strategy is to calculate the reduction vectors that are used to construct the observables from the node activities. These vectors fully determine the reduced approximate dynamics, including a reduced adjacency matrix that specifies the magnitude of the coupling between observables.

We described two methods for computing the reduction vectors. In what we call the *homogeneous reduction*, the observables are obtained from homogeneously averaging the activities within the different groups of nodes. The approximated reduced dynamics on these observables has the same form as the original dynamics. Also, the reduced adjacency matrix is such that the interaction from group $G_{\rho }$ to group $G_{\nu }$ is given by the average weighted in-degree that nodes in $G_{\nu }$ receive from nodes in $G_{\rho }$. This corresponds to what a naive observer would do to coarse-grain the original system.

Systems that are highly heterogeneous or for which it is difficult to define proper node partitions will not typically be well reduced by such a homogeneous coarse-graining. The main result of the present work is the definition of another procedure to construct the observables which can better cope with heterogeneities in the structure of interactions. In what we dub the *spectral reduction*, the reduction vectors are no longer homogeneous over the nodes that form each of the different groups. Instead, they weigh the nodes differently so as to minimize the error of the approximated reduced dynamics. Finding the reduction vectors in this case requires solving a set of compatibility equations on these vectors. Despite the compatibility equations being generically incompatible, we proposed an algorithm for finding an approximate solution when the adjacency matrix is positive. The resulting approximate reduced dynamics is analogous in form to the original system except for the addition of a correction term.

We verified that both the homogeneous and the spectral reduction are suitable for reducing systems in which a proper node partition is identified and nodes in the same group have very similar connectivity profiles. In hindsight, this may come as no surprise. Indeed, a system composed of groups of nearly equivalent nodes can be naively reduced by identifying each group with its activity average; the averages then approximately obey the same dynamical laws as the original system with interaction strengths that come from averaging the weighted in-degrees from the different groups. This is exactly what our homogeneous reduction does. In these homogeneous networks with properly identified community structure, the spectral reduction provides almost no gain with respect to the homogeneous one.

We then analyzed the performance of the spectral reduction in more heterogeneous directed networks with known community structure. We found that the spectral reduction outperforms the homogeneous one and that it can be used to effectively reduce the dimension of these systems when a proper node partition is defined. The quality of the reduction can be enhanced by refining the partition so as to reduce the heterogeneity of nodes within each group. Doing so of course increases the reduced dimension *n* and, with it, the complexity of the reduced dynamics. Our results suggest, however, that it is possible to find a compromise between increasing the number of groups and obtaining a reduced system of dimension significantly smaller than the original one because, unlike the homogeneous reduction, the spectral reduction can cope with a certain degree of inter-group heterogeneity. Similar results are obtained when real networks are analyzed, even if we do not know what the true community structure, if any, is. Provided that a prior study of their structure is performed to identify possible communities, the spectral reduction could contribute to understand and identify relevant features in the behavior of real complex systems.

However, classifying nodes into groups of similar connectivity profiles can be a difficult task. Even if different algorithms to this end are currently available, a search for communities will generally be imperfect, either because there are aspects of network architecture that can be difficult to extract or because a modular structure does not really exist in such networks. The presence of communities is often just an abstract notion that helps us to dissect and understand networks, but that fits to a network’s structure only vaguely. For this reason, we would like our reduction method to be not too sensitive to errors in the definition of the node partition. We discovered that the spectral reduction is quite robust to partition perturbations, which makes it a good candidate to reduce systems in which communities cannot be well defined or identified.

Compared to other work on this topic, the dimension reduction strategy we have presented offers some important advantages. First, it can be applied to systems in which the adjacency matrix is weighted and not necessarily symmetric (i.e. directed networks). Second, the dimension of the reduced dynamics, *n*, is a variable that can be chosen to better adapt the reduction to the particular system under study. This feature adds flexibility to the reduction and can be very useful when dealing with systems of different degrees of complexity. The dimension of a proper reduction also provides information about the effective dimension of the system under study, and this information would be more difficult to infer if the dimension of the reduction were fixed a priori. Finally, since our observables are weighted averages of the node activities within the different groups, they have a very clear interpretation: each observable represents the activity within a group (this feature is not obvious or even possible in other dimension-reduction methods where the reduction vectors can have positive and negative entries across all the node indices).

How the activity within a group is weighted is specified by the entries of the reduction vectors. The reduction vectors are found by (approximately) solving the compatibility equations, which are necessary to (approximately) close the reduced dynamics. When the original adjacency matrix is positive, the compatibility equations can be rewritten as a set of decoupled equations for the different reduction vectors. For a given group index ν, the equations involving the group-to-group adjacency matrices are


(20) $$\begin{array}{cc} W_{\nu \nu }^{T}{\hat{a}}_{\nu } & =\lambda_{\nu \nu }^{\prime }{\hat{a}}_{\nu }\\ W_{\rho \nu }^{T}W_{\nu \rho }^{T}{\hat{a}}_{\nu } & =\lambda_{\nu \rho }^{\prime }{\hat{a}}_{\nu },\;\nu \neq \rho , \end{array}$$

so ${\hat{a}}_{\nu }$ should be the dominant eigenvector (normalized to have sum 1) of $W_{\nu \nu }^{T}$ and $W_{\rho \nu }^{T}W_{\nu \rho }^{T}$ for all ρ≠ν. Matrix $W_{\nu \nu }$ specifies the weights of the paths of length 1 connecting two nodes in $G_{\nu }$; matrix $W_{\nu \rho }W_{\rho \nu }$ specifies the sum of the weights of all the paths of length 2 connecting two nodes in $G_{\nu }$ via nodes in $G_{\rho }$. Thus, both $W_{\nu \nu }$ and $W_{\nu \rho }W_{\rho \nu }$ can be seen as interaction matrices among nodes in $G_{\nu }$, the first taking into account direct interactions and the second taking into account 2-step interactions through nodes in $G_{\rho }$. The entries of the normalized dominant eigenvector of a positive interaction matrix are a measure of the “importance” of the nodes in the corresponding network: the so-called *eigenvector centrality* (55). Thus, Eq. 20 state that the contribution of a given node in $G_{\nu }$ to the corresponding observable $X_{\nu }$ should be given by this centrality measure. When the eigenvector centrality of a node is different on each of these 1-step and 2-step matrices (i.e. when Eq. 20 do not have an exact solution), we propose to find an approximate solution that is as close as possible to the different centralities.

The compatibility equations thus suggest that, in order to make the reduced system be as close as possible to the real observables’ dynamics, the contribution of each node to its corresponding observable has to be the relative centrality of the node within that group, where this centrality should take into account both direct (1-step) and indirect (2-step) interactions via nodes in other groups. This is clearly shown by a limit example in which there is a subset of nodes that are completely disconnected from the network (and thus they do not play any role in the network’s dynamics): the spectral reduction assigns zero weight to these nodes when they are packed in a group *G* that also contains a connected node (and the reduction is exact when each of the remaining groups is composed of a single node, see Proposition 3 in the Supplementary material). In the same example, the homogeneous reduction would assign the same weight to all the nodes in *G*, giving a far from accurate reduced dynamics because it would not be constructed based on any notion of the nodes’ relevance in the network.

The reduced system not only allows us to approximate critical parameters at which the system’s behavior qualitatively changes, but provides us with a direct measure of how the activity within these groups will be affected by a parameter perturbation around the critical value. For example, in a system modeling an ecological dynamics, our observables reflect the overall abundance of different groups of species, so by studying the reduced system we can infer which species groups could go extinct as a parameter is perturbed.

Our work leaves also some open questions. To construct the spectral reduction, one has to approximately solve the compatibility equations on the reduction vectors. We have proposed a method for doing so when the adjacency matrix is positive, but other procedures should be defined to deal with general types of interactions. In neuronal networks, for example, inhibition plays a crucial role along with excitation, but so far our method can only be applied to networks composed exclusively of excitatory or inhibitory units. Another limitation of the present work is that it is still unclear how to find the dimension *n* of a proper reduction without comparing the reduced system with the original one. Since the performance of the reduction is tightly related to the properties of the chosen partition—i.e. the number of groups and the intra-group heterogeneity—it is likely that some measure on the network’s structure under the chosen partition can be used to predict how accurate the reduced dynamics will be. Elucidating these and other issues will require additional research in the coming years.

## Supplementary Material

pgad150_Supplementary_Data

## Data Availability

The code used to produce all figures is publicly available on Zenodo (https://doi.org/10.5281/zenodo.7632921).

## Preprints

This manuscript was posted on a preprint: https://doi.org/10.48550/arXiv.2206.11230.
