# Supplementary material for: Dimension reduction of dynamics on modular and heterogeneous directed networks
Source: PNAS Nexus. 2023 May 2;2(5):pgad150. doi: 10.1093/pnasnexus/pgad150 (PMC10198746; doi:10.1093/pnasnexus/pgad150)
Supplement: pgad150_Supplementary_Data [file pgad150_supplementary_data.zip › PNASNEXUS-PNASNEXUS-2022-01018-s01.pdf]

Dimension reduction of dynamics on modular and heterogeneous directed networks

— Supplementary Information —

Preliminaries

We start by presenting some preliminary assumptions and definitions.

Assumption 1 (Complete system) For $N \in \mathbb{N}$, there are N activity functions $x_1, \cdots, x_N$ of class $\mathscr{C}^2$ with

$$\begin{array}{rccc} x_i : & \mathbb{R} & \to & \mathbb{R} \\ & t & \mapsto & x_i(t). \end{array} \tag{S1}$$

These functions fulfill the system of ODEs

$$\dot{x}_i = f(x_i) + \sum_{j=1}^{N} w_{ij}\, g(x_i, x_j), \qquad i \in \{1, \cdots, N\}, \tag{S2}$$

where $f : \mathbb{R} \to \mathbb{R}$, $g : \mathbb{R}^2 \to \mathbb{R}$ are functions of class $\mathscr{C}^1$, and w_{ij} denotes a real number for all $i, j \in \{1, \cdots, N\}$.

Definition 1 (Adjacency and in-degree matrices) Given a system under Assumption 1, we call $\boldsymbol{W} = (w_{ij})_{i,j=1}^N$ the *adjacency matrix*. The latter defines a weighted directed network of N nodes and M links, where M is the number of nonzero elements in $\boldsymbol{W}$. We define the *in-degree matrix* as the $N \times N$ diagonal matrix constructed from the weighted in-degrees of the nodes in the network, that is,

$$\boldsymbol{K} = \mathrm{diag}(k_1, \cdots, k_N), \quad k_i = \sum_{j=1}^{N} w_{ij}. \tag{S3}$$

Assumption 2 (Reduced system) Given a system defined by Assumption 1, there is a $n \in \mathbb{N}$, $n \leq N$, and a partition of the nodes into n non-empty groups $G_1, \cdots, G_n$, that is, $G_\nu \neq \varnothing$ for all ν, $\cup_{\nu=1}^n G_\nu = \{1, \cdots, N\}$, and $G_\nu \cap G_\rho = \varnothing$ for $\nu \neq \rho$. There is also a set of n reduction vectors $\boldsymbol{a}_1, \cdots, \boldsymbol{a}_n$ where $\boldsymbol{a}_\nu = (a_{\nu i})_{i=1}^N \in \mathbb{R}^N$ is the reduction vector associated to group G_ν, and

$$\sum_{i=1}^{N} a_{\nu i} = 1 \tag{S4}$$

$$a_{\nu i} = 0 \ \text{ if } \ i \notin G_\nu \tag{S5}$$

for all $\nu \in \{1, \cdots, n\}$. There are n observables $\mathcal{X}_1, \cdots, \mathcal{X}_n$ of class $\mathscr{C}^1$ with

$$\begin{array}{rccc} \mathcal{X}_\nu : & \mathbb{R} & \to & \mathbb{R} \\ & t & \mapsto & \mathcal{X}_\nu(t) \end{array} \tag{S6}$$

which are constructed from the reduction vectors and the activity functions through

$$\mathcal{X}_\nu := \sum_{i=1}^{N} a_{\nu i} x_i, \quad \nu \in \{1, \cdots, n\}. \tag{S7}$$

Assumption 3 (Ordered partition) Let $\{G_1, \cdots, G_n\}$ be a partition of $\{1, \cdots, N\}$, that is, $G_\nu \neq \varnothing$ for all ν, $\cup_{\nu=1}^n G_\nu = \{1, \cdots, N\}$, and $G_\nu \cap G_\rho = \varnothing$ for $\nu \neq \rho$. The indices within each partition set are consecutive integers:

$$G_\nu = \left\{ 1 + \sum_{\rho=1}^{\nu-1} m_\rho, \cdots, \sum_{\rho=1}^{\nu} m_\rho \right\}, \tag{S8}$$

where $m_\nu = |G_\nu|$ is the size of G_ν. Each index in $\{1, \cdots, m_\nu\}$ is mapped to an index in $G_\nu \subseteq \{1, \cdots, N\}$ by

$$\begin{array}{rccl} p_\nu : & \{1, \cdots, m_\nu\} & \to & G_\nu \\ & i & \mapsto & p_\nu(i) := i + \sum_{\rho=1}^{\nu-1} m_\rho. \end{array} \tag{S9}$$

Without loss of generality we can always suppose that Assumption 3 holds when dealing with a system defined by Assumptions 1 and 2 (it is enough to permute the node indices $1, \cdots, N$). This allows us to express the adjacency and in-degree matrices as follows.

Definition 2 (Group-to-group interaction and in-degree matrices) Under Assumptions 1 and 3, for each pair of indices $\nu, \rho \in \{1, \cdots, n\}$, we define the submatrix of interactions from G_ρ to G_ν, $\boldsymbol{W}_{\nu\rho} = ([\boldsymbol{W}_{\nu\rho}]_{ij})_{i,j}$, of dimension $m_\nu \times m_\rho$, and the in-degree submatrix of nodes in G_ν from nodes in G_ρ, $\boldsymbol{K}_{\nu\rho} = \mathrm{diag}([\boldsymbol{K}_{\nu\rho}]_{ii})_i$, of dimension $m_\nu \times m_\nu$, as

$$\begin{aligned} [\boldsymbol{W}_{\nu\rho}]_{ij} &= w_{p_\nu(i)p_\rho(j)} \\ [\boldsymbol{K}_{\nu\rho}]_{ii} &= \sum_{j \in G_\rho} w_{p_\nu(i)j} = k^\rho_{p_\nu(i)} \end{aligned} \tag{S10}$$

for $i \in \{1, \cdots, m_\nu\}$ and $j \in \{1, \cdots, m_\rho\}$, where $k^\rho_{p_\nu(i)}$ is the weighted in-degree of node $p_\nu(i)$ from G_ρ. This allows to express the adjacency matrix $\boldsymbol{W}$ in the block form

$$\boldsymbol{W} = \begin{pmatrix} \boldsymbol{W}_{11} & \cdots & \boldsymbol{W}_{1n} \\ \vdots & \ddots & \vdots \\ \boldsymbol{W}_{n1} & \cdots & \boldsymbol{W}_{nn} \end{pmatrix} \tag{S11}$$

and the in-degree matrix as

$$\boldsymbol{K} = \begin{pmatrix} \boldsymbol{K}_{11} + \cdots + \boldsymbol{K}_{1n} & \cdots & 0 \\ \vdots & \ddots & \vdots \\ 0 & \cdots & \boldsymbol{K}_{n1} + \cdots + \boldsymbol{K}_{nn} \end{pmatrix}. \tag{S12}$$

It is also useful to define, for each $\nu \in \{1, \cdots, n\}$, the components of the reduction vector $\boldsymbol{a}_\nu$ that correspond to the indices within G_ν (the other components are zero):

Definition 3 (Partial reduction vectors) Under Assumptions 2 and 3, for each $\nu \in \{1, \cdots, n\}$, the ν-th *partial reduction vector* $\widehat{\boldsymbol{a}}_\nu = (\widehat{a}_{\nu i})_{i=1}^{m_\nu} \in \mathbb{R}^{m_\nu}$ is such that

$$\widehat{a}_{\nu i} := a_{\nu\, p_\nu(i)}, \qquad i \in \{1, \dots, m_\nu\}. \tag{S13}$$

Notice that the normalization condition still holds on $\widehat{\boldsymbol{a}}_\nu$: $\sum_{i=1}^{m_\nu} \widehat{a}_{\nu i} = 1$.

Approximate reduced dynamics

Here we describe two possible strategies for reducing the dimension of a system defined by Assumption 1 by means of the observables presented in Assumption 2. In both cases, we approximate $f(x_i)$ and $g(x_i, x_j)$ by Taylor polynomials around the observables associated to the groups to which i and j belong. The reason for this is that nodes will be partitioned into groups so that nodes in the same group have similar connectivity properties. If the partition is well chosen, the activities of nodes in the same group should be close to each other and also close to the corresponding observable. The two strategies result in what we have dubbed the *homogeneous* and the *spectral* reductions. We first provide a lemma and some useful notation.

Lemma 1 (Exact reduced dynamics) *Under Assumptions 1 and 2, the observables fulfill the system of ODEs*

$$\dot{\mathcal{X}}_\nu = \sum_{i=1}^N a_{\nu i} f(x_i) + \sum_{i,j=1}^N a_{\nu i} w_{ij} g(x_i, x_j), \qquad \nu \in \{1, \dots, n\}. \tag{S14}$$

Proof The result follows directly from Assumptions 1 and 2. □

Notation 1 (Big $\mathcal{O}$) Let $g : \mathbb{R}^2 \to \mathbb{R}$, $h : \mathbb{R}^4 \to \mathbb{R}$ be two functions. Let $x, X \in \mathbb{R}$, $\boldsymbol{x} = (x_1, x_2)^T$, $\boldsymbol{X} = (X_1, X_2)^T \in \mathbb{R}^2$. We write

$$g(x, X) = \mathcal{O}\left(x - X\right) \tag{S15}$$

whenever there exists $K \in \mathbb{R}$ such that

$$\lim_{x \to X} \frac{g(x, X)}{x - X} = K. \tag{S16}$$

We write

$$h(x_1, x_2, X_1, X_2) = \mathcal{O}\left((x_1 - X_1)(x_2 - X_2)\right) \tag{S17}$$

whenever there exists $L \in \mathbb{R}$ such that

$$\lim_{\|\boldsymbol{x} - \boldsymbol{X}\| \to 0} \frac{h(x_1, x_2, X_1, X_2)}{(x_1 - X_1)(x_2 - X_2)} = L. \tag{S18}$$

Notation 2 (Function approximation) Let $f : \mathbb{R}^{\mathbb{N}} \to \mathbb{R}$, $F : \mathbb{R}^n \to \mathbb{R}$ be two functions. Given $n \leq N$, let $\{G_1, \cdots, G_n\}$ be a partition of $\{1, \cdots, N\}$. For $\boldsymbol{x} \in \mathbb{R}^N$ and $\boldsymbol{X} \in \mathbb{R}^n$, we write

$$f(\boldsymbol{x}) \overset{\mathcal{O}_1}{\approx} F(\boldsymbol{X}) \tag{S19}$$

whenever

$$f(\boldsymbol{x}) - F(\boldsymbol{X}) = \sum_{\nu=1}^{n} \sum_{i \in G_\nu} \mathcal{O}\left(x_i - X_\nu\right). \tag{S20}$$

We write

$$f(\boldsymbol{x}) \overset{\mathcal{O}_2}{\approx} F(\boldsymbol{X}) \tag{S21}$$

whenever

$$f(\boldsymbol{x}) - F(\boldsymbol{X}) = \sum_{\nu,\rho=1}^{n} \sum_{\substack{i \in G_\nu \\ j \in G_\rho}} \mathcal{O}\left((x_i - X_\nu)(x_j - X_\rho)\right). \tag{S22}$$

Remark 1 *For the sake of notational simplicity, we have not included the dependence of* $\mathcal{O}_1$ *and* $\mathcal{O}_2$ *upon the partition* $\{G_1, \cdots, G_n\}$. *It should be understood however that the* $\mathcal{O}_1$ *and* $\mathcal{O}_2$ *approximations are much more restrictive than the usual first and second-order approximations. Indeed,* $f(\boldsymbol{x}) \overset{\mathcal{O}_1}{\approx} F(\boldsymbol{X})$ *if and only if, in the general expansion*

$$f(\boldsymbol{x}) - F(\boldsymbol{X}) = \sum_{\nu=1}^{n} \sum_{i=1}^{N} c_{\nu i}\, \mathcal{O}\left(x_i - X_\nu\right), \tag{S23}$$

the coefficients $c_{\nu i}$ *are exactly zero whenever* $i \notin G_\nu$. *Similarly,* $f(\boldsymbol{x}) \overset{\mathcal{O}_2}{\approx} F(\boldsymbol{X})$ *if and only if, in the general expansion*

$$f(\boldsymbol{x}) - F(\boldsymbol{X}) = \sum_{\nu,\rho=1}^{n} \sum_{i,j=1}^{N} c_{\nu\rho ij}\, \mathcal{O}\left((x_i - X_\nu)(x_j - X_\rho)\right), \tag{S24}$$

the coefficients $c_{\nu\rho ij}$ *are exactly zero whenever* $i \notin G_\nu$ *or* $j \notin G_\rho$. *Thus, general first and second-order approximations respectively have up to* nN *and* n^2N^2 *error terms, while the* $\mathcal{O}_1$ *and* $\mathcal{O}_2$ *approximations can only lead to* N *and* N^2 *error terms, respectively.*

Now, let us assume that we are given a network and that the node partition is performed adequately. If each variable X_ν *is a weighted average as in Eq.* (S7)*, then the absolute value of* $x_i - X_\nu$ *should be small for all* $i \in G_\nu$. *Therefore, we expect* $f(\boldsymbol{x}) \overset{\mathcal{O}_1}{\approx} F(\boldsymbol{X})$ *and* $f(\boldsymbol{x}) \overset{\mathcal{O}_2}{\approx} F(\boldsymbol{X})$ *to provide good approximations since in both cases,* $f(\boldsymbol{x}) - F(\boldsymbol{X})$ *only contains small correction terms like* $x_i - X_\nu$*, where* $i \in G_\nu$*, without any contribution from potentially large factors such as* $x_i - X_\rho$*, where* $i \notin G_\rho$.

With the above notation in hand, we can present the homogeneous and the spectral reductions. Both reductions are induced by reduction vectors that depend explicitly on a node partition and satisfy an additional group-exclusivity constraint, namely Eq. (S5).

Lemma 2 (Group-exclusive reduction) *Suppose that Assumptions 1 and 2 hold. Then*

$$\dot{\mathcal{X}}_\nu \overset{\mathcal{O}_1}{\approx} f(\mathcal{X}_\nu) + \sum_{\rho=1}^{n} \mathcal{W}_{\nu\rho}\, g(\mathcal{X}_\nu, \mathcal{X}_\rho), \tag{S25}$$

where

$$\mathcal{W}_{\nu\rho} := \sum_{\substack{i \in G_\nu \\ j \in G_\rho}} a_{\nu i} w_{ij} = \sum_{i \in G_\nu} a_{\nu i} k_i^\rho. \tag{S26}$$

Proof By Taylor's Theorem, we can approximate $f(x_i)$ and $g(x_i, x_j)$ around the corresponding observables as

$$\begin{aligned} f(x_i) &\overset{\mathcal{O}_1}{\approx} f(\mathcal{X}_\nu) && \text{for } i \in G_\nu \\ g(x_i, x_j) &\overset{\mathcal{O}_1}{\approx} g(\mathcal{X}_\nu, \mathcal{X}_\rho) && \text{for } i \in G_\nu, j \in G_\rho. \end{aligned} \tag{S27}$$

Taking into account that, by construction, $a_{\nu k} = 0$ whenever $k \notin G_\nu$, from Lemma 1 we get

$$\begin{aligned} \dot{\mathcal{X}}_\nu &\overset{\mathcal{O}_1}{\approx} f(\mathcal{X}_\nu) \sum_{i \in G_\nu} a_{\nu i} + \sum_{\rho=1}^{n} g(\mathcal{X}_\nu, \mathcal{X}_\rho) \sum_{\substack{i \in G_\nu \\ j \in G_\rho}} a_{\nu i} w_{ij} \\ &= f(\mathcal{X}_\nu) + \sum_{\rho=1}^{n} \mathcal{W}_{\nu\rho}\, g(\mathcal{X}_\nu, \mathcal{X}_\rho), \end{aligned} \tag{S28}$$

where we have used the normalization condition Eq. (S4) to go get the second equality. ☐

Remark 2 *The essential condition to get the approximate dynamical system Eq.* (S25) *is the group-exclusivity of the reduction vectors, namely Eq.* (S5). *Without group-exclusivity, reaching* $\mathcal{O}_1$ *approximation is impossible in general. Reduction vectors that do not satisfy Eq.* (S5) *would still lead to a first-order approximation, but the corresponding error function would contain considerably more terms than the* $\mathcal{O}_1$ *approximation (see Remark 1).*

The normalization condition Eq. (S4) *has no fundamental role; it only helps simplifying the resulting equations. For instance, without Eq.* (S4)*, the reduced system in Eq.* (S25) *would contain a* ν*-dependent coefficient multiplying* $f(\mathcal{X}_\nu)$*.*

Corollary 1 (Homogeneous reduction) Suppose that Assumptions 1 and 2 hold. If

$$a_{\nu i} = \begin{cases} 1/|G_\nu| & \text{if } i \in G_\nu, \\ 0 & \text{otherwise,} \end{cases} \tag{S29}$$

then

$$\dot{\mathcal{X}}_\nu \overset{\mathcal{O}_1}{\approx} f(\mathcal{X}_\nu) + \sum_{\rho=1}^{n} \mathcal{W}_{\nu\rho}\, g(\mathcal{X}_\nu, \mathcal{X}_\rho), \tag{S30}$$

where $\mathcal{W}_{\nu\rho}$ is the average weighted in-degree received by group ν and coming from group ρ, i.e.,

$$\mathcal{W}_{\nu\rho} := \frac{1}{|G_\nu|} \sum_{i \in G_\nu} k_i^\rho. \tag{S31}$$

Proof This follows immediately form the previous lemma and Eq. (S29). □

We have thus proved that all group-exclusive reduction vectors, including the group-homogeneous reduction vectors satisfying Eq. (S29), lead to $\mathcal{O}_1$–approximated reduced dynamical systems. To improve these approximations and reach the $\mathcal{O}_2$ level, additional conditions must be imposed on the reduction vectors. Below, we prove that if these vectors satisfy eigenvalue equations coming from the group-to-group interaction and in-degree matrices, then the observables $\mathcal{X}_1, \ldots, \mathcal{X}_n$ evolve according to a $\mathcal{O}_2$-approximated system of n ODEs.

Proposition 1 (Spectral reduction) *Suppose that Assumptions 1, 2 and 3 hold. Let* $\{\boldsymbol{W}_{\nu\rho}\}_{\nu,\rho}$, $\{\boldsymbol{K}_{\nu\rho}\}_{\nu,\rho}$ *and* $\{\widehat{\boldsymbol{a}}_\nu\}_\nu$ *be the sets of matrices and vectors of Definitions 2 and 3. If there exist two matrices* $\boldsymbol{\mu} = (\mu_{\nu\rho})_{\nu,\rho}$ *and* $\boldsymbol{\lambda} = (\lambda_{\nu\rho})_{\nu,\rho}$ *of dimension* $n \times n$ *such that the partial reduction vectors fulfill the compatibility equations*

$$\boldsymbol{K}_{\nu\rho}\widehat{\boldsymbol{a}}_\nu = \mu_{\nu\rho}\widehat{\boldsymbol{a}}_\nu, \tag{S32a}$$

$$\boldsymbol{W}_{\nu\rho}^T\widehat{\boldsymbol{a}}_\nu = \lambda_{\nu\rho}\widehat{\boldsymbol{a}}_\rho, \tag{S32b}$$

then

$$\dot{\mathcal{X}}_\nu \overset{\mathcal{O}_2}{\approx} f(\mathcal{X}_\nu) + \sum_{\rho=1}^{n} \mathcal{W}_{\nu\rho}\, g(\mathcal{X}_\nu, \mathcal{X}_\rho), \tag{S33}$$

where

$$\mathcal{W}_{\nu\rho} := \sum_{\substack{i \in G_\nu \\ j \in G_\rho}} a_{\nu i} w_{ij} = \sum_{i \in G_\nu} a_{\nu i} k_i^\rho. \tag{S34}$$

Proof By Taylor's Theorem we can approximate $f(x_i)$ and $g(x_i, x_j)$ at first order around the corresponding observables as

$$\begin{aligned} f(x_i) &\overset{\mathcal{O}_2}{\approx} f(\mathcal{X}_\nu) + f'(\mathcal{X}_\nu)(x_i - \mathcal{X}_\nu) && \text{for } i \in G_\nu, \\ g(x_i, x_j) &\overset{\mathcal{O}_2}{\approx} g(\mathcal{X}_\nu, \mathcal{X}_\rho) + g_1(\mathcal{X}_\nu, \mathcal{X}_\rho)(x_i - \mathcal{X}_\nu) + g_2(\mathcal{X}_\nu, \mathcal{X}_\rho)(x_j - \mathcal{X}_\rho) && \text{for } i \in G_\nu, j \in G_\rho. \end{aligned} \tag{S35}$$

We rewrite Eq. (S14) as

$$\begin{aligned} \dot{\mathcal{X}}_\nu &= \sum_{i \in G_\nu} a_{\nu i} f(x_i) + \sum_{\rho=1}^{n} \sum_{\substack{i \in G_\nu \\ j \in G_\rho}} a_{\nu i} w_{ij} g(x_i, x_j) \\ &= T_\nu + \sum_{\rho=1}^{n} T_{\nu\rho}, \end{aligned} \tag{S36}$$

where

$$\begin{aligned} T_\nu &:= \sum_{i \in G_\nu} a_{\nu i} f(x_i), \\ T_{\nu\rho} &:= \sum_{\substack{i \in G_\nu \\ j \in G_\rho}} a_{\nu i} w_{ij} g(x_i, x_j). \end{aligned} \tag{S37}$$

Using approximation (S35), the definition of observables and the normalization condition on $\boldsymbol{a}_\nu$, $\sum_{i\in G_\nu} a_{\nu i} = 1$, we obtain the following first-order approximations:

$$\begin{aligned} T_\nu &\overset{\mathcal{O}_2}{\approx} f(\mathcal{X}_\nu) + f'(\mathcal{X}_\nu) \sum_{i\in G_\nu} a_{\nu i}(x_i - \mathcal{X}_\nu) \\ &= f(\mathcal{X}_\nu), \end{aligned} \tag{S38a}$$

$$\begin{aligned} T_{\nu\rho} &\overset{\mathcal{O}_2}{\approx} g(\mathcal{X}_\nu, \mathcal{X}_\rho) \sum_{\substack{i\in G_\nu \\ j\in G_\rho}} a_{\nu i} w_{ij} + g_1(\mathcal{X}_\nu, \mathcal{X}_\rho) \sum_{\substack{i\in G_\nu \\ j\in G_\rho}} a_{\nu i} w_{ij}(x_i - \mathcal{X}_\nu) + g_2(\mathcal{X}_\nu, \mathcal{X}_\rho) \sum_{\substack{i\in G_\nu \\ j\in G_\rho}} a_{\nu i} w_{ij}(x_j - \mathcal{X}_\rho) \\ &= \mathcal{W}_{\nu\rho}\, g(\mathcal{X}_\nu, \mathcal{X}_\rho) + g_1(\mathcal{X}_\nu, \mathcal{X}_\rho) \left(\sum_{\substack{i\in G_\nu \\ j\in G_\rho}} a_{\nu i} w_{ij} x_i - \mathcal{W}_{\nu\rho}\mathcal{X}_\nu \right) + g_2(\mathcal{X}_\nu, \mathcal{X}_\rho) \left(\sum_{\substack{i\in G_\nu \\ j\in G_\rho}} a_{\nu i} w_{ij} x_j - \mathcal{W}_{\nu\rho}\mathcal{X}_\rho \right). \end{aligned} \tag{S38b}$$

We can rewrite the compatibility equations in component form as follows:

$$[\boldsymbol{K}_{\nu\rho}]_{ii}\, \widehat{a}_{\nu i} = \mu_{\nu\rho} \widehat{a}_{\nu i} \qquad i \in \{1, \cdots, m_\nu\}, \tag{S39a}$$

$$\sum_{i=1}^{m_\nu} [\boldsymbol{W}_{\nu\rho}]_{ij} \widehat{a}_{\nu i} = \lambda_{\nu\rho} \widehat{a}_{\rho j} \qquad j \in \{1, \cdots, m_\rho\}. \tag{S39b}$$

This can in turn be expressed as a function of the general interaction and degree matrices and the complete reduction vectors as

$$\sum_{j\in G_\rho} w_{ij} a_{\nu i} = \mu_{\nu\rho} a_{\nu i} \qquad i \in G_\nu, \tag{S40a}$$

$$\sum_{i\in G_\nu} w_{ij} a_{\nu i} = \lambda_{\nu\rho} a_{\rho j} \qquad j \in G_\rho. \tag{S40b}$$

Given an arbitrary activity vector $\boldsymbol{x} = (x_1, \cdots, x_N)^T$, we now multiply both sides of Eq. (S40a) by x_i and we sum over $i \in G_\nu$. We also multiply both sides of Eq. (S40b) by x_j and we sum over $j \in G_\rho$. We get

$$\sum_{\substack{i\in G_\nu \\ j\in G_\rho}} w_{ij} a_{\nu i} x_i = \mu_{\nu\rho} \sum_{i\in G_\nu} a_{\nu i} x_i = \mu_{\nu\rho} \mathcal{X}_\nu \tag{S41a}$$

$$\sum_{\substack{i\in G_\nu \\ j\in G_\rho}} w_{ij} a_{\nu i} x_j = \lambda_{\nu\rho} \sum_{j\in G_\rho} a_{\rho j} x_j = \lambda_{\nu\rho} \mathcal{X}_\rho. \tag{S41b}$$

On the other hand, we can sum Eq. (S40a) over $i \in G_\nu$ and Eq. (S40b) over $j \in G_\rho$ to get

$$\mathcal{W}_{\nu\rho} = \sum_{\substack{i\in G_\nu \\ j\in G_\rho}} w_{ij} a_{\nu i} = \mu_{\nu\rho} \sum_{i\in G_\nu} a_{\nu i} = \mu_{\nu\rho} \tag{S42a}$$

$$\mathcal{W}_{\nu\rho} = \sum_{\substack{i\in G_\nu \\ j\in G_\rho}} w_{ij} a_{\nu i} = \lambda_{\nu\rho} \sum_{j\in G_\rho} a_{\rho j} = \lambda_{\nu\rho}. \tag{S42b}$$

Plugging Eqs. (S41a), (S41b), (S42a) and (S42b) into Eqs. (S38a) and (S38b) we get

$$T_\nu \overset{\mathcal{O}_2}{\approx} f(\mathcal{X}_\nu) \tag{S43a}$$

$$T_{\nu\rho} \overset{\mathcal{O}_2}{\approx} \mathcal{W}_{\nu\rho}\, g(\mathcal{X}_\nu, \mathcal{X}_\rho), \tag{S43b}$$

which completes the proof. □

Notice that, given a set of vectors $\widehat{\boldsymbol{a}}_1, \cdots, \widehat{\boldsymbol{a}}_n$, the matrices of scalars $\boldsymbol{\mu} = (\mu_{\nu\rho})_{\nu,\rho}$, $\boldsymbol{\lambda} = (\lambda_{\nu\rho})_{\nu,\rho}$ that solve or minimize the quadratic error in the compatibility equations (S32a), (S32b) are given by

$$\mu_{\nu\rho} = \frac{\widehat{\boldsymbol{a}}_\nu^T \boldsymbol{K}_{\nu\rho} \widehat{\boldsymbol{a}}_\nu}{\|\widehat{\boldsymbol{a}}_\nu\|^2} \tag{S44a}$$

$$\lambda_{\nu\rho} = \frac{\widehat{\boldsymbol{a}}_\rho^T \boldsymbol{W}_{\nu\rho}^T \widehat{\boldsymbol{a}}_\nu}{\|\widehat{\boldsymbol{a}}_\rho\|^2} \tag{S44b}$$

(see Lemma 3 below).

Lemma 3 *Let $\boldsymbol{M}$ be a matrix of dimension $r \times s$ and let $\boldsymbol{u}$, $\boldsymbol{v} \neq \boldsymbol{0}$ be vectors of dimension s and r, respectively. The scalar λ that minimizes*

$$\|\boldsymbol{M}\boldsymbol{u} - \lambda \boldsymbol{v}\|^2 \tag{S45}$$

is given by

$$\lambda = \boldsymbol{v}^{+}\boldsymbol{M}\boldsymbol{u}, \tag{S46}$$

where

$$\boldsymbol{v}^{+} = \frac{1}{\|\boldsymbol{v}\|^2}\boldsymbol{v}^T \tag{S47}$$

is the Moore-Penrose pseudoinverse of the vector $\boldsymbol{v}$.

Proof Define the function $F : \mathbb{R} \to \mathbb{R}$ as

$$F(\lambda) = \|\boldsymbol{M}\boldsymbol{u} - \lambda \boldsymbol{v}\|^2. \tag{S48}$$

This function is obviously smooth. Moreover, it is convex since $F''(\lambda) = 2\langle \boldsymbol{v}, \boldsymbol{v}\rangle > 0$. The minimum of F is thus found by solving $F'(\lambda) = 0$. However,

$$F'(\lambda) = -2\langle \boldsymbol{v}, \boldsymbol{M}\boldsymbol{u} - \lambda\boldsymbol{v}\rangle = -2\left(\boldsymbol{v}^T\boldsymbol{M}\boldsymbol{u} - \lambda\boldsymbol{v}^T\boldsymbol{v}\right), \tag{S49}$$

which is zero whenever $\boldsymbol{v}^T\boldsymbol{M}\boldsymbol{u} = \lambda\boldsymbol{v}^T\boldsymbol{v}$. Therefore, the minimum of F is defined by

$$\lambda = \frac{\boldsymbol{v}^T\boldsymbol{M}\boldsymbol{u}}{\boldsymbol{v}^T\boldsymbol{v}}, \tag{S50}$$

which in turn is equivalent to Eq. (S46) since $\boldsymbol{v}^{+}$, the Moore-Penrose pseudoinverse of $\boldsymbol{v}$, is equal to $\boldsymbol{v}^T/(\boldsymbol{v}^T\boldsymbol{v}) = \boldsymbol{v}^T/\|\boldsymbol{v}\|^2$. □

Proposition 2 (Homogeneous modular network) *Suppose that Assumptions 1, 2 and 3 hold. Let $\{\boldsymbol{W}_{\nu\rho}\}_{\nu,\rho}$, $\{\boldsymbol{K}_{\nu\rho}\}_{\nu,\rho}$ and $\{\widehat{\boldsymbol{a}}_\nu\}_\nu$ be the sets of matrices and vectors of Definitions 2 and 3. If the connectivity structure is modular and perfectly homogeneous according to groups $G_1, \cdots, G_n$, that is, if the group-to-group interaction matrices have the form*

$$\boldsymbol{W}_{\nu\rho} = w_{\nu\rho}\begin{pmatrix} 1 & \cdots & 1 \\ \vdots & & \vdots \\ 1 & \cdots & 1 \end{pmatrix}, \qquad w_{\nu\rho} \in \mathbb{R}, \quad \nu, \rho \in \{1, \cdots, n\},$$

then there exists a unique solution to the compatibility equations (S32a), (S32b) *and it is given by*

$$\widehat{\boldsymbol{a}}_\nu = \frac{1}{m_\nu}(1, \cdots, 1)^T \in \mathbb{R}^{m_\nu}, \qquad \nu \in \{1, \cdots, n\}.$$

Moreover, the homogeneous and the spectral reductions defined by Corrolary 1 and Proposition 1 coincide, with

$$\mathcal{W}_{\nu\rho} = w_{\nu\rho}m_\rho.$$

Proof The weighted in-degree of an arbitrary node i in group G_ν from G_ρ is $k_i^\rho = w_{\nu\rho}m_\rho$, so the group-to-group in-degree matrices have the form

$$\boldsymbol{K}_{\nu\rho} = w_{\nu\rho}m_\rho \,\mathrm{diag}(1, \cdots, 1), \qquad \nu, \rho \in \{1, \cdots, n\}.$$

In particular, any vector in $\mathbb{R}^{m_\nu}$ is an eigenvector of $\boldsymbol{K}_{\nu\rho}$ (of eigenvalue $w_{\nu\rho}m_\rho$) and Eq. (S32a) is fulfilled for any vector $\widehat{\boldsymbol{a}}_\nu$ whenever $\mu_{\nu\rho} = w_{\nu\rho}m_\rho$. In the proof of Proposition 1 we have shown that when the compatibility equations (S32a), (S32b) are fulfilled, the scalars in these equations coincide: $\mu_{\nu\rho} = \lambda_{\nu\rho}$ for all $\nu, \rho \in \{1, \cdots, n\}$. Thus, in our case $\lambda_{\nu\rho} = w_{\nu\rho}m_\rho$. Also, the left-hand-side of Eq. (S32b) is

$$\boldsymbol{W}_{\nu\rho}^T\widehat{\boldsymbol{a}}_\nu = w_{\nu\rho}\begin{pmatrix} 1 & \cdots & 1 \\ \vdots & & \vdots \\ 1 & \cdots & 1 \end{pmatrix}\widehat{\boldsymbol{a}}_\nu = w_{\nu\rho}\left(\sum_{i=1}^{m_\nu}\widehat{\boldsymbol{a}}_{\nu i}\right)\begin{pmatrix} 1 \\ \vdots \\ 1 \end{pmatrix},$$

so if the vector set $\{\widehat{\boldsymbol{a}}_\nu\}_{\nu=1}^n$, with $\sum_{i=1}^{m_\nu}\widehat{\boldsymbol{a}}_{\nu i} = 1$ for all ν, is a solution to Eq. (S32b) with $\lambda_{\nu\rho} = w_{\nu\rho}m_\rho$, then

$$\boldsymbol{W}_{\nu\rho}^T\widehat{\boldsymbol{a}}_\nu = w_{\nu\rho}\begin{pmatrix} 1 \\ \vdots \\ 1 \end{pmatrix} = w_{\nu\rho}m_\rho\frac{1}{m_\rho}\begin{pmatrix} 1 \\ \vdots \\ 1 \end{pmatrix} = \lambda_{\nu\rho}\frac{1}{m_\rho}\begin{pmatrix} 1 \\ \vdots \\ 1 \end{pmatrix} = \lambda_{\nu\rho}\widehat{\boldsymbol{a}}_\rho$$

and necessarily $\widehat{\boldsymbol{a}}_\rho = \frac{1}{m_\rho}(1, \cdots, 1)^T$ for all $\rho \in \{1, \cdots, n\}$. Therefore, the homogeneous and spectral reductions coincide, with

$$\mathcal{W}_{\nu\rho} = \lambda_{\nu\rho} = \mu_{\nu\rho} = w_{\nu\rho}m_\rho.$$

□

Proposition 3 (Network with disconnected nodes) *Suppose that Assumptions 1 and 2 hold. If the nodes in $S = \{1, \cdots, k\}$ are disconnected from all the other nodes in the network, then the spectral reduction of dimension $n = N - k$ associated to the node partition*

$$G_1 = S \cup \{k+1\}, \quad G_2 = \{k+2\}, \quad \cdots, \quad G_{N-k} = \{N\}$$

is exact and has partial reduction vectors

$$\widehat{\boldsymbol{a}}_1 = (0, \cdots, 0, 1) \in \mathbb{R}^{k+1}, \quad \widehat{\boldsymbol{a}}_\rho = (1) \quad \forall \rho \in \{2, \cdots, N-k\}.$$

Proof We first show that the compatibility equations have a unique exact solution. The group-to-group adjacency matrices are

$$\boldsymbol{W}_{11} = \begin{pmatrix} w_1 & \cdots & 0 & 0 \\ \vdots & \ddots & \vdots & \vdots \\ 0 & \cdots & w_k & 0 \\ 0 & \cdots & 0 & w_{11} \end{pmatrix}, \quad \boldsymbol{W}_{1\rho} = \begin{pmatrix} 0 \\ \vdots \\ 0 \\ w_{1\rho} \end{pmatrix}, \quad \boldsymbol{W}_{\rho 1} = \begin{pmatrix} 0 & \cdots & 0 & w_{\rho 1} \end{pmatrix}, \quad \boldsymbol{W}_{\nu\rho} = (w_{\nu\rho}) \quad \forall \nu, \rho \neq 1,$$

where $w_1, \cdots, w_k \in \mathbb{R}$ are the weights of the self-connections of nodes in S and $w_{\nu\rho} \in \mathbb{R}$ is the weight of the connection from node $k+\rho$ to node $k+\nu$. The group-to-group weighted in-degree matrices are

$$\boldsymbol{K}_{11} = \boldsymbol{W}_{11}, \quad \boldsymbol{K}_{1\rho} = \operatorname{diag}(0, \cdots, 0, w_{1\rho}), \quad \boldsymbol{K}_{\rho 1} = (w_{\rho 1}), \quad \boldsymbol{K}_{\nu\rho} = (w_{\nu\rho}) \quad \forall \nu, \rho \neq 1.$$

The partial reduction vector associated to any group $G_\rho \neq G_1$ is simply $\widehat{\boldsymbol{a}}_\rho = (1)$ because G_ρ contains a single node. It is straightforward to see that the compatibility equations involving the partial reduction vectors of the groups different from G_1 are then automatically fulfilled. The remaining partial reduction vector, $\widehat{\boldsymbol{a}}_1$, has to fulfill the remaining compatibility equations,

$$\boldsymbol{K}_{1\rho}\widehat{\boldsymbol{a}}_1 = \mu_{1\rho}\widehat{\boldsymbol{a}}_1 \tag{S51a}$$

$$\boldsymbol{W}_{1\rho}^T\widehat{\boldsymbol{a}}_1 = \lambda_{1\rho}\widehat{\boldsymbol{a}}_\rho \tag{S51b}$$

$$\boldsymbol{W}_{\rho 1}^T\widehat{\boldsymbol{a}}_\rho = \lambda_{\rho 1}\widehat{\boldsymbol{a}}_1 \tag{S51c}$$

for all ρ. For $\rho \neq 1$, imposing Eq. (S51c) we have

$$\boldsymbol{W}_{\rho 1}^T\widehat{\boldsymbol{a}}_\rho = \begin{pmatrix} 0 \\ \vdots \\ 0 \\ w_{\rho 1} \end{pmatrix} (1) = \begin{pmatrix} 0 \\ \vdots \\ 0 \\ w_{\rho 1} \end{pmatrix} \overset{\text{(S51c)}}{=} \lambda_{\rho 1}\,\widehat{\boldsymbol{a}}_1,$$

so

$$\widehat{\boldsymbol{a}}_1 = (0, \cdots, 0, 1)^T$$

(the reduction vectors must have sum 1) and $\lambda_{\rho 1} = w_{\rho 1}$. It is easy to check that this solution fulfills all the other equations with $\lambda_{1\rho} = \mu_{1\rho} = w_{1\rho}$ and $\lambda_{\rho 1} = \mu_{\rho 1} = w_{\rho 1}$. Thus, the compatibility equations have a unique and exact solution.

We now show that the resulting spectral reduction is exact. Due to the specific form of the reduction vectors that solve the compatibility equations,

$$\mathcal{W}_{\nu\rho} = w_{\nu\rho} \quad \text{and} \quad \mathcal{X}_\nu = x_{k+\nu} \quad \forall \nu, \rho \in \{1, \cdots, n\},$$

and the exact reduced dynamics is

$$\dot{\mathcal{X}}_\nu = f(x_{k+\nu}) + \sum_{j=1}^{N} w'_{(k+\nu)j}\, g(x_{k+\nu}, x_j), \qquad \nu \in \{1, \ldots, n\}, \tag{S52}$$

where $w'_{(k+\nu)j} = 0$ for $j \in S$ and $w'_{(k+\nu)j} = w_{\nu(j-k)}$ for $j \notin S$. This becomes

$$\begin{aligned} \dot{\mathcal{X}}_\nu &= f(x_{k+\nu}) + \sum_{j=k+1}^{N} w'_{(k+\nu)j}\, g(x_{k+\nu}, x_j) \\ &= f(x_{k+\nu}) + \sum_{\rho=1}^{n} w_{\nu\rho}\, g(x_{k+\nu}, x_{k+\rho}) \\ &= f(\mathcal{X}_\nu) + \sum_{\rho=1}^{n} \mathcal{W}_{\nu\rho}\, g(\mathcal{X}_\nu, \mathcal{X}_\rho), \qquad \nu \in \{1, \ldots, n\}, \end{aligned} \tag{S53}$$

so the reduced dynamics is exact. □

Correction when the compatibility equations cannot be solved exactly

The compatibility equations (S32a)–(S32b) provide sufficient conditions for obtaining an approximate reduced system that remains valid up to second order. However, in general, these equations cannot be fulfilled simultaneously. As detailed in the next sections, we can circumvent this problem by prioritizing Eq. (S32b) when solving the compatibility equations. This means that we find a matrix $\boldsymbol{\lambda} = (\lambda_{\nu\rho})_{\nu,\rho}$ and vectors $\widehat{\boldsymbol{a}}_1, \cdots, \widehat{\boldsymbol{a}}_n$ that approximately fulfill Eq. (S32b) but not necessarily Eq. (S32a). Then, for all $\nu, \rho \in \{1, \cdots, n\}$, we set $\mu_{\nu\rho}$ to be the parameter that minimizes the quadratic error of Eq. (S32a), that is,

$$\mu_{\nu\rho} = \widehat{\boldsymbol{a}}_\nu^+ \boldsymbol{K}_{\nu\rho} \widehat{\boldsymbol{a}}_\nu = \frac{\widehat{\boldsymbol{a}}_\nu^T \boldsymbol{K}_{\nu\rho} \widehat{\boldsymbol{a}}_\nu}{\|\widehat{\boldsymbol{a}}_\nu\|^2} \tag{S54}$$

(see Lemma 3). We can thus reasonably assume that Eqs. (S41b) and (S42b) hold. We also assume that Eqs. (S41a) hold —otherwise the observables' dynamics cannot be expressed in a closed form— but not necessarily Eqs. (S42a), so that the approximate reduced dynamics has an additional correction term:

$$\dot{\mathcal{X}}_\nu \approx f(\mathcal{X}_\nu) + \sum_{\rho=1}^{n} \mathcal{W}_{\nu\rho}\, g(\mathcal{X}_\nu, \mathcal{X}_\rho) + \sum_{\rho=1}^{n} g_1(\mathcal{X}_\nu, \mathcal{X}_\rho)\,(\mu_{\nu\rho} - \mathcal{W}_{\nu\rho})\,\mathcal{X}_\nu \tag{S55}$$

with $\{\mathcal{W}_{\nu\rho}\}_{\nu,\rho}$ as defined in Proposition 1 by Eq. (S34). Whenever Eqs. (S42a) hold, $\mathcal{W}_{\nu\rho} = \mu_{\nu\rho}$ for all ν, ρ and we recover the reduced dynamics of Proposition 1.

Equivalent forms for the compatibility equations when the adjacency matrix is positive

Here we show that, whenever the adjacency matrix $\boldsymbol{W}$ is positive and we want the vectors $\widehat{\boldsymbol{a}}_1, \cdots, \widehat{\boldsymbol{a}}_n$ to be also positive, we can transform the compatibility equations into another set of equivalent, decoupled equations. We start by presenting some useful propositions.

Proposition 4 Let $\boldsymbol{A}$, $\boldsymbol{B}$ be two positive matrices of dimension $n \times m$ and $m \times n$, respectively. Let $\boldsymbol{u}$, $\boldsymbol{v}$ be two non-zero vectors of dimension n and m, respectively. Then:

1. There exists a scalar $\lambda > 0$ that is a dominant eigenvalue of both matrices $\boldsymbol{AB}$ and $\boldsymbol{BA}$ (that is, any other eigenvalue λ' of $\boldsymbol{AB}$ or $\boldsymbol{BA}$ is smaller in modulus: $|\lambda'| < \lambda$). The multiplicity of λ as an eigenvalue of both $\boldsymbol{AB}$ and $\boldsymbol{BA}$ is 1.
2. If $\boldsymbol{u}$ and $\boldsymbol{v}$ are, respectively, eigenvectors of $\boldsymbol{AB}$ and $\boldsymbol{BA}$ associated to the dominant eigenvalue λ, then there exist scalars $\alpha, \beta \neq 0$ such that
$$\begin{aligned} \boldsymbol{Av} &= \alpha \boldsymbol{u} \\ \boldsymbol{Bu} &= \beta \boldsymbol{v}. \end{aligned} \tag{S56}$$

Proof

1. $\boldsymbol{AB}$ and $\boldsymbol{BA}$ are positive matrices. Then, the Perron-Frobenius Theorem states that they have dominant eigenvalues λ and λ', respectively, that are positive and have multiplicity 1. We only need to prove that $\lambda = \lambda'$. Let $\boldsymbol{u}$ be an eigenvector of $\boldsymbol{AB}$ associated to λ:
$$\boldsymbol{ABu} = \lambda \boldsymbol{u}.$$
Left-multiplying this equation by $\boldsymbol{B}$ we have
$$\boldsymbol{BA}(\boldsymbol{Bu}) = \lambda \boldsymbol{Bu}.$$
$\boldsymbol{Bu} \neq \boldsymbol{0}$ because otherwise we would have $\boldsymbol{0} = \boldsymbol{ABu} = \lambda \boldsymbol{u}$ and this is absurd since λ and $\boldsymbol{u}$ are non-zero. Therefore, $\boldsymbol{Bu}$ is an eigenvector of $\boldsymbol{BA}$ with eigenvalue λ, which implies $\lambda \leq \lambda'$. Inverting the roles of $\boldsymbol{A}$ and $\boldsymbol{B}$ we obtain $\lambda' \leq \lambda$. We conclude that $\lambda = \lambda'$.
2. Let $\boldsymbol{u}$ and $\boldsymbol{v}$ be, respectively, eigenvectors of $\boldsymbol{AB}$ and $\boldsymbol{BA}$ associated to the dominant eigenvalue λ. In the proof of point 1. we have shown that $\boldsymbol{Bu}$ and $\boldsymbol{Av}$ are eigenvectors of $\boldsymbol{BA}$ and $\boldsymbol{AB}$, respectively, with eigenvalue λ. We also know that λ has multiplicity 1 in both cases, which means that the eigenspaces associated to λ for $\boldsymbol{AB}$ and $\boldsymbol{BA}$ have dimension 1. Therefore, $\boldsymbol{Bu}$ and $\boldsymbol{Av}$ have to be multiples of $\boldsymbol{v}$ and $\boldsymbol{u}$, respectively: there exist scalars $\alpha, \beta \neq 0$ such that
$$\begin{aligned} \boldsymbol{Av} &= \alpha \boldsymbol{u} \\ \boldsymbol{Bu} &= \beta \boldsymbol{v}. \end{aligned}$$

□

Proposition 5 Let $\boldsymbol{A}$, $\boldsymbol{B}$ be two positive matrices of dimension $n \times m$ and $m \times n$, respectively. Let $\boldsymbol{u}$, $\boldsymbol{v}$ be two vectors of dimension n and m, respectively, such that all their entries are positive. Then, the following statements are equivalent:

1. There exist scalars $\alpha, \beta > 0$ such that
$$\begin{aligned} \boldsymbol{Av} &= \alpha \boldsymbol{u} \\ \boldsymbol{Bu} &= \beta \boldsymbol{v}. \end{aligned} \tag{S57}$$

2. There exists a scalar $\lambda > 0$ such that

$$\begin{aligned} \boldsymbol{ABu} &= \lambda\boldsymbol{u} \\ \boldsymbol{BAv} &= \lambda\boldsymbol{v}. \end{aligned} \tag{S58}$$

Proof We start by showing that 1. implies 2. If we left-multiply the first equality in Eq. (S57) by $\boldsymbol{B}$ and then use the second one, we get

$$\boldsymbol{BAv} = \alpha\boldsymbol{Bu} = \alpha\beta\boldsymbol{v}. \tag{S59}$$

Analogously,

$$\boldsymbol{ABu} = \beta\boldsymbol{Av} = \alpha\beta\boldsymbol{u}. \tag{S60}$$

Therefore, 2. holds with $\lambda = \alpha\beta > 0$.

Let us show now that 2. implies 1. From 2. we have that $\boldsymbol{u}$ and $\boldsymbol{v}$ are, respectively, eigenvectors of $\boldsymbol{AB}$ and $\boldsymbol{BA}$ with eigenvalue λ. Since all the components of $\boldsymbol{u}$ and $\boldsymbol{v}$ are positive and $\boldsymbol{AB}$, $\boldsymbol{BA}$ are positive matrices, by the Perron-Frobenius Theorem they must be dominant eigenvectors of $\boldsymbol{AB}$ and $\boldsymbol{BA}$. This means that λ is the dominant eigenvalue of both matrices. Thus, according to Proposition 4, there exist scalars $\alpha, \beta \neq 0$ such that Eq. (S57) holds. Moreover, α, β are positive because $\boldsymbol{AB}, \boldsymbol{BA}$ are positive matrices and $\boldsymbol{u}, \boldsymbol{v}$ have positive entries. □

Notice that going from 1. to 2. is straightforward and does not require the positiveness hypothesis on $\boldsymbol{A}$, $\boldsymbol{B}$, $\boldsymbol{u}$ and $\boldsymbol{v}$. These conditions are nonetheless needed to deduce 1. from 2. The following corollary is a direct application of Proposition 5 to the compatibility equations.

Corollary 2 For $\nu, \rho \in \{1, \cdots, n\}$, let $\boldsymbol{W}_{\nu\rho}$ be a positive matrix of dimension $m_\nu \times m_\rho$ and let $\widehat{\boldsymbol{a}}_\nu$ be a positive vector of dimension m_ν. Then, the following statements are equivalent:

1. There exists a set of positive scalars $\{\lambda_{\nu\rho}\}_{\nu,\rho}$ such that

$$\begin{aligned} \boldsymbol{W}_{\nu\nu}^T\widehat{\boldsymbol{a}}_\nu &= \lambda_{\nu\nu}\widehat{\boldsymbol{a}}_\nu, & \nu &\in \{1, \cdots, n\} \\ \boldsymbol{W}_{\nu\rho}^T\widehat{\boldsymbol{a}}_\nu &= \lambda_{\nu\rho}\widehat{\boldsymbol{a}}_\rho, & \nu, \rho &\in \{1, \cdots, n\},\ \nu \neq \rho. \end{aligned} \tag{S61}$$

2. There exists a set of positive scalars $\{\lambda'_{\nu\rho}\}_{\nu,\rho}$ such that

$$\begin{aligned} \boldsymbol{W}_{\nu\nu}^T\widehat{\boldsymbol{a}}_\nu &= \lambda'_{\nu\nu}\widehat{\boldsymbol{a}}_\nu, & \nu &\in \{1, \cdots, n\} \\ \boldsymbol{W}_{\rho\nu}^T\boldsymbol{W}_{\nu\rho}^T\widehat{\boldsymbol{a}}_\nu &= \lambda'_{\nu\rho}\widehat{\boldsymbol{a}}_\nu, & \nu, \rho &\in \{1, \cdots, n\},\ \nu \neq \rho. \end{aligned} \tag{S62}$$

Moreover, the relation between the scalars of statements 1. and 2. is

$$\begin{aligned} \lambda'_{\nu\nu} &= \lambda_{\nu\nu} \\ \lambda'_{\nu\rho} &= \lambda_{\nu\rho}\lambda_{\rho\nu} \qquad \text{for } \nu \neq \rho. \end{aligned} \tag{S63}$$

This allows us to transform the original compatibility equations, Eqs. (S32a)–(S32b), into the *decoupled compatibility equations*

$$\boldsymbol{K}_{\nu\rho}\widehat{\boldsymbol{a}}_\nu = \mu_{\nu\rho}\widehat{\boldsymbol{a}}_\nu \tag{S64a}$$

$$\boldsymbol{W'}_{\nu\rho}\widehat{\boldsymbol{a}}_\nu = \lambda'_{\nu\rho}\widehat{\boldsymbol{a}}_\nu, \tag{S64b}$$

where

$$\lambda'_{\nu\rho} := \begin{cases} \lambda_{\nu\nu} & \text{if } \nu = \rho \\ \lambda_{\nu\rho}\lambda_{\rho\nu} & \text{if } \nu \neq \rho \end{cases}, \qquad \boldsymbol{W'}_{\nu\rho} := \begin{cases} \boldsymbol{W}_{\nu\nu}^T & \text{if } \nu = \rho \\ \boldsymbol{W}_{\rho\nu}^T\boldsymbol{W}_{\nu\rho}^T & \text{if } \nu \neq \rho \end{cases}. \tag{S65}$$

Once Eqs. (S64a)–(S64b) are solved and $\widehat{\boldsymbol{a}}_\nu$ is known for all ν, the original set of compatibility equations (S32a)–(S32b) is fulfilled and the set of scalars $\{\mu_{\nu\rho}, \lambda_{\nu\rho}\}_{\nu,\rho}$ can be determined via Eqs. (S42a)–(S42b) or (S44a)–(S44b).

As we have noticed after the proof of the second result, the positiveness of $\boldsymbol{W}_{\rho\nu}^T$ and $\widehat{\boldsymbol{a}}_\nu$ for all ν, ρ is needed to obtain the equivalence between Eqs. (S61) and Eqs. (S62). If we relax this hypothesis, solving the decoupled equations might not be sufficient for solving the original set of equations.

The decoupled compatibility equations (S64a)–(S64b) are not simultaneously solvable in general. In the next sections we present a possible strategy to find an approximate solution.

An approximate solution to the compatibility equations that involve the adjacency matrix

Let us focus on approximately solving Eqs. (S64b) for a fixed ν and variable ρ. For this, we assume that the scalars $\lambda'_{\nu 1}, \cdots, \lambda'_{\nu n}$ are the dominant eigenvalues of the matrices involved (this would be the case if Eqs. (S64b) could be solved *exactly*). Our goal then is to find a vector that has sum 1 and minimizes the sum of the corresponding quadratic errors. For now we will relax the condition of the vector having positive entries. We can formulate the problem as follows:

Problem 1 *Given a set of $m \times m$ positive matrices $\{\boldsymbol{M}_i\}_{i=1}^n$ and scalars $\{\lambda_i\}_{i=1}^n$, find a vector $\boldsymbol{a} = (a_i)_{i=1}^m \in \mathbb{R}^m$ with $\sum_{i=1}^m a_i = 1$ and such that the following error is minimal:*

$$E(\boldsymbol{a}) := \|\boldsymbol{M}_1\boldsymbol{a} - \lambda_1\boldsymbol{a}\|^2 + \cdots + \|\boldsymbol{M}_n\boldsymbol{a} - \lambda_n\boldsymbol{a}\|^2. \tag{S66}$$

Proposition 6 *Let $\boldsymbol{u}_1, \cdots, \boldsymbol{u}_r$ be r linearly independent, non-negative vectors in $\mathbb{R}^m$. Without loss of generality we can assume that these vectors are normalized: $\sum_{i=1}^m [\boldsymbol{u}_s]_i = 1$ for all $s \in \{1, \cdots, r\}$. Then, a solution $\boldsymbol{a} \in \mathbb{R}^m$ to Problem 1 of the form*

$$\boldsymbol{a}(\boldsymbol{x}) := x_1\boldsymbol{u}_1 + \cdots + x_r\boldsymbol{u}_r \tag{S67}$$

must satisfy the following condition: the vector $\boldsymbol{y} := (x_1, \cdots, x_r, K)^T$, for some non-zero constant K, is a solution to the system of $r+1$ linear equations

$$\hat{\boldsymbol{C}}\boldsymbol{y} \;=\; (0, \cdots, 0, 1)^T \tag{S68}$$

where $\hat{\boldsymbol{C}} = \left(\begin{array}{c|c} \boldsymbol{C} & \boldsymbol{-1} \\ \hline \boldsymbol{1}^T & 0 \end{array}\right)$ and $\boldsymbol{C} = (c_{st})_{s,t}$ is the $r \times r$ matrix defined by

$$c_{st} := \sum_{j=1}^n \langle \boldsymbol{M}_j\boldsymbol{u}_s - \lambda_j\boldsymbol{u}_s, \boldsymbol{M}_j\boldsymbol{u}_t - \lambda_j\boldsymbol{u}_t\rangle. \tag{S69}$$

Proof We are looking for a solution $\boldsymbol{a}$ within the subspace spanned by $\boldsymbol{u}_1, \cdots, \boldsymbol{u}_r$. The vector $\boldsymbol{a}$ can thus be expressed as

$$\boldsymbol{a}(\boldsymbol{x}) = x_1\boldsymbol{u}_1 + \cdots + x_r\boldsymbol{u}_r \tag{S70}$$

for some $\boldsymbol{x} = (x_1, \cdots, x_r)^T$. Since $\sum_{i=1}^m [\boldsymbol{u}_s]_i = 1$ by assumption, the condition $\sum_{i=1}^m a_i = 1$ requires $\sum_{i=1}^r x_i = 1$. We thus have to find $\boldsymbol{x}$ such that $\boldsymbol{a}(\boldsymbol{x})$ minimizes $E(\boldsymbol{a}(\boldsymbol{x}))$ subject to the constraint

$$\sum_{i=1}^r x_i = 1. \tag{S71}$$

This is a minimization problem with a single constraint that can be solved using the method of the Lagrange multipliers: considering the Lagrangian function

$$\mathcal{L}(\boldsymbol{x}, K) := \sum_{j=1}^n \|\boldsymbol{M}_j\boldsymbol{a}(\boldsymbol{x}) - \lambda_j\boldsymbol{a}(\boldsymbol{x})\|^2 + 2K\left(1 - \sum_{s=1}^r x_s\right) = E(\boldsymbol{a}(\boldsymbol{x})) + 2K\left(1 - \sum_{s=1}^r x_s\right), \tag{S72}$$

a local solution to the problem necessarily fulfills

$$\frac{\partial}{\partial x_i}\mathcal{L}(\boldsymbol{x}, K) = 0 \quad \forall i, \qquad \frac{\partial}{\partial K}\mathcal{L}(\boldsymbol{x}, K) = 0. \tag{S73}$$

Let us notice that, for a given $j \in \{1, \cdots, n\}$,

$$\begin{aligned}
\|\boldsymbol{M}_j\boldsymbol{a}(\boldsymbol{x}) - \lambda_j\boldsymbol{a}(\boldsymbol{x})\|^2 &= \|\sum_{s=1}^r x_s\left(\boldsymbol{M}_j\boldsymbol{u}_s - \lambda_j\boldsymbol{u}_s\right)\|^2 \\
&= \langle\sum_{s=1}^r x_s\left(\boldsymbol{M}_j\boldsymbol{u}_s - \lambda_j\boldsymbol{u}_s\right), \sum_{t=1}^r x_t\left(\boldsymbol{M}_j\boldsymbol{u}_t - \lambda_j\boldsymbol{u}_t\right)\rangle \\
&= \sum_{s,t=1}^r x_s x_t \langle\boldsymbol{M}_j\boldsymbol{u}_s - \lambda_j\boldsymbol{u}_s, \boldsymbol{M}_j\boldsymbol{u}_t - \lambda_j\boldsymbol{u}_t\rangle \\
&= \sum_{s,t=1}^r x_s x_t\, c_{st}^j \\
&= \langle\boldsymbol{x}, \boldsymbol{C}_j\boldsymbol{x}\rangle,
\end{aligned} \tag{S74}$$

where $\langle\cdot,\cdot\rangle$ denotes the scalar product and we have defined

$$\boldsymbol{C}_j := (c_{st}^j)_{s,t}, \qquad c_{st}^j := \langle\boldsymbol{M}_j\boldsymbol{u}_s - \lambda_j\boldsymbol{u}_s, \boldsymbol{M}_j\boldsymbol{u}_t - \lambda_j\boldsymbol{u}_t\rangle. \tag{S75}$$

Due to the symmetry of the scalar product, $\boldsymbol{C}_j$ is a symmetric matrix for all j. Introducing the matrix $\boldsymbol{C} := \sum_{j=1}^{n} \boldsymbol{C}_j$ we have

$$\begin{aligned} \mathcal{L}(\boldsymbol{x}, K) &= \sum_{j=1}^{n} \langle \boldsymbol{x}, \boldsymbol{C}_j \boldsymbol{x} \rangle + 2K \left(1 - \sum_{s=1}^{r} x_s\right) \\ &= \langle \boldsymbol{x}, \boldsymbol{C}\boldsymbol{x} \rangle + 2K \left(1 - \sum_{s=1}^{r} x_s\right) \end{aligned} \tag{S76}$$

and

$$\begin{aligned} \frac{\partial}{\partial x_i} \mathcal{L}(\boldsymbol{x}, K) &= \langle \frac{\partial}{\partial x_i} \boldsymbol{x}, \boldsymbol{C}\boldsymbol{x} \rangle + \langle \boldsymbol{x}, \boldsymbol{C} \frac{\partial}{\partial x_i} \boldsymbol{x} \rangle - 2K \\ &= 2 \langle \frac{\partial}{\partial x_i} \boldsymbol{x}, \boldsymbol{C}\boldsymbol{x} \rangle - 2K \\ &= 2\left([\boldsymbol{C}\boldsymbol{x}]_i - K\right), \end{aligned} \tag{S77}$$

where in the second equality we have taken into account that $\boldsymbol{C}$ is symmetric. We can finally express the set of equations (S73) as

$$\begin{aligned} \boldsymbol{C}\boldsymbol{x} &= K\boldsymbol{1} \\ \sum_{s=1}^{r} x_s &= 1, \end{aligned} \tag{S78}$$

where $\boldsymbol{1} = (1, \cdots, 1)^T$. This is a system of $r + 1$ linear equations that can in turn be rewritten as

$$\hat{\boldsymbol{C}} \boldsymbol{y} = (0, \cdots, 0, 1)^T \tag{S79}$$

with $\hat{\boldsymbol{C}} := \left(\begin{array}{c|c} \boldsymbol{C} & -\boldsymbol{1} \\ \hline \boldsymbol{1}^T & 0 \end{array}\right)$ and $\boldsymbol{y} := (x_1, \cdots, x_r, K)$. □

We observe the following:

- *The error associated to a solution* $(\boldsymbol{x}, K)$ *is given by* K. If $(\boldsymbol{x}, K)$ is a solution to Eq. (S78), then the error $E(\boldsymbol{a}(\boldsymbol{x}))$ and the Lagrangian function $\mathcal{L}(\boldsymbol{x}, K)$ take the same value and this is

$$\begin{aligned} E(\boldsymbol{a}(\boldsymbol{x})) &= \langle \boldsymbol{x}, \boldsymbol{C}\boldsymbol{x} \rangle \\ &= \langle \boldsymbol{x}, K\boldsymbol{1} \rangle \\ &= K. \end{aligned} \tag{S80}$$

- *All the solutions to Eq.* (S78) *produce the same error.* Suppose, on the contrary, that there are two solutions, $s_1 = (\boldsymbol{x}_1, K_1)$, $s_2 = (\boldsymbol{x}_2, K_2)$ that produce different errors, that is, with $K_1 \neq K_2$. Any point in the segment that connects s_1 and s_2, $s(h) = (1-h)s_1 + hs_2$, $h \in (0, 1)$, is also a solution and its corresponding Lagrangian function is $\mathcal{L}(s(h)) = (1-h)K_1 + hK_2$. Since $K_1 \neq K_2$, this means that the directional derivative of the Lagrangian function along this segment is non-zero for all h, which contradicts the fact that the solutions to Eq. (S78) are the points for which all the directional derivatives of the Lagrangian function are zero. We conclude that $K_1 = K_2$.

We can apply this result to our set of decoupled compatibility equations that involve the adjacency matrix. If we do not impose that $\widehat{\boldsymbol{a}}_\nu$ has positive entries and we assume that it lies in the subspace spanned by a collection of r vectors $\boldsymbol{u}_1, \cdots, \boldsymbol{u}_r \in \mathbb{R}^{m_\nu}$ with $\sum_{i=1}^{m_\nu} [\boldsymbol{u}_s]_i = 1$ for all s, then

$$\widehat{\boldsymbol{a}}_\nu = x_1 \boldsymbol{u}_1 + \cdots + x_r \boldsymbol{u}_r \tag{S81}$$

and the $x_1, \cdots, x_r$ parameters are obtained by solving Eq. (S68) with matrix $\boldsymbol{C}$ defined by

$$c_{st} := \sum_{\rho=1}^{n} \langle \boldsymbol{W}'_{\nu\rho} \boldsymbol{u}_s - \lambda'_{\nu\rho} \boldsymbol{u}_s, \boldsymbol{W}'_{\nu\rho} \boldsymbol{u}_t - \lambda'_{\nu\rho} \boldsymbol{u}_t \rangle. \tag{S82}$$

A solution that is not restricted to a particular subspace is obtained when $r = m_\nu$ and $\boldsymbol{u}_1, \cdots, \boldsymbol{u}_{m_\nu}$ is the canonical basis of $\mathbb{R}^{m_\nu}$. This solution is the one with the smallest error. We call it the *optimal* solution.

However, it can be useful to seek a solution in a subspace of dimension $r \ll m_\nu$ because the associated system of linear equations will be of smaller dimension and, therefore, easier and faster to solve. Taking into account that the parameters $\lambda'_{\nu 1}, \cdots, \lambda'_{\nu n}$ are in fact the dominant eigenvalues of matrices $\boldsymbol{W}'_{\nu 1}, \cdots, \boldsymbol{W}'_{\nu n}$, we hypothesized that the optimal solution is close to the subspace spanned by the dominant eigenvectors of these matrices. This is clearly the case when $n = 1$ and when the matrices share their dominant eigenspace.

To test this hypothesis, we randomly generated sets of n matrices of dimension $m \times m$ and we compared the optimal solution with the solution that is restricted to the subspace spanned by the dominant eigenvectors, for different choices of n and m. The results show that the error associated to the restricted solution is only slightly larger than that of the optimal solution (Fig. S1A) and that the two solutions are very similar (Fig. S1B), which suggests that the solution that is restricted to the subspace of dominant eigenvectors is a good approximation to the optimal solution. As expected, the two solutions coincide whenever $n = m$.

In the results shown here we restrict the solution to Eq. (S68) to the subspace spanned by the dominant eigenvectors. We have observed that, despite not explicitly requiring $\widehat{\boldsymbol{a}}_\nu$ to be a positive vector, this is so in all the cases studied.

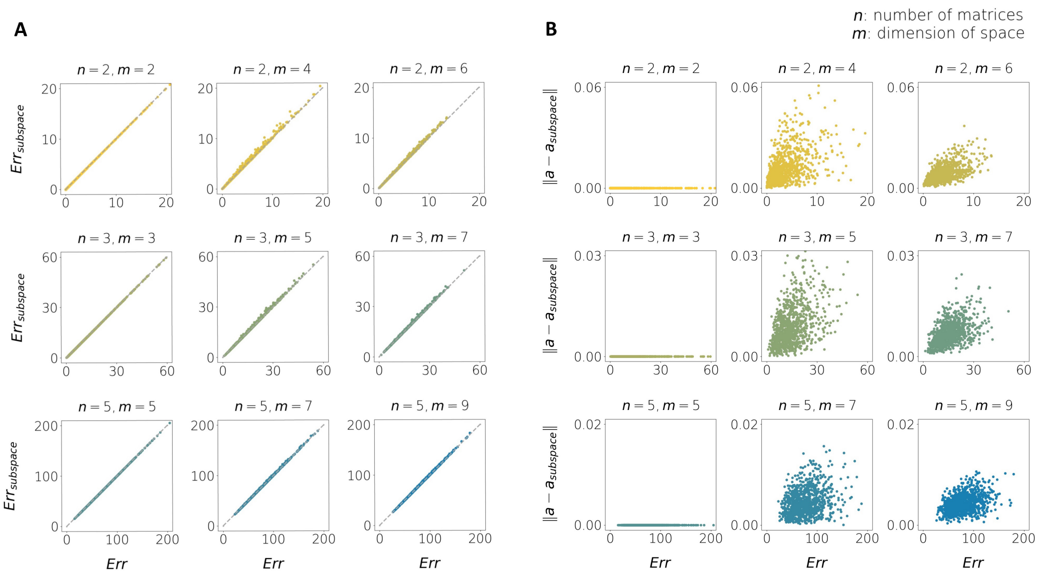

Fig. S1. Comparison of the optimal and restricted solutions to Problem 1, defined by Eqs. (S67)), (S68), the restricted solution being the one that lies in the subspace spanned by the dominant eigenvectors of the matrices involved. For each choice of n (number of matrices) and m (dimension of space), we randomly generated 1000 sets of $m \times m$ matrices, we found their dominant eigenvalues and both solutions were computed. For a fixed n, the elements of the i-th matrix were created independently within the range $(0, 1+5i)$, $i \in \{1, \cdots, n\}$. **A**. Error associated to the restricted solution (Err_{subspace}) versus error associated to the optimal solution (Err). **B**. Euclidean distance between the two solutions ($\boldsymbol{a}$, $\boldsymbol{a}_{\text{subspace}}$) versus error associated to the optimal solution.

Construction of heterogeneous networks with communities

A heterogeneous network with block structure and in/out-degree variability is constructed as follows. First, a partition of the nodes into n groups is defined. Let m_ν be the size of group G_ν. For every ordered pair of group indices (ν, ρ), a parameter $p_{\nu\rho}$ defines the mean connection density of interactions from G_ρ to G_ν. Every node i in G_ν is assigned a collection of hidden in/out-degrees from/to the other groups: $\boldsymbol{\kappa}^{\boldsymbol{i},\mathbf{in}} = (\kappa_1^{i,\text{in}}, \cdots, \kappa_n^{i,\text{in}})$, $\boldsymbol{\kappa}^{\boldsymbol{i},\mathbf{out}} = (\kappa_1^{i,\text{out}}, \cdots, \kappa_n^{i,\text{out}})$ such that

$$\begin{aligned} \langle \kappa_\rho^{i,\text{in}} \rangle &= m_\rho \, p_{\nu\rho} \\ \langle \kappa_\rho^{i,\text{out}} \rangle &= m_\rho \, p_{\rho\nu}. \end{aligned} \tag{S83}$$

Once the hidden degrees are specified, a connection from node $j \in G_\rho$ to node $i \in G_\nu$ is created with probability

$$p_{ij} = \frac{\kappa_\rho^{i,\text{in}} \, \kappa_\nu^{j,\text{out}}}{m_\nu \, m_\rho \, p_{\nu\rho}}. \tag{S84}$$

The hidden degrees can follow any distribution provided that their expectation is the one specified above and that the hidden degrees of every node are independent of those of any other node. We can also incorporate a correlation between the hidden in- and out-degrees of single nodes. In our example networks they are uniformly distributed and the hidden in/out-degrees of a node from/to its own group are correlated with correlation coefficient $\rho_{\text{in/out}} = 0.8$.

Partition refinement

Given a network and a node partition, we refine the partition (that is, we split the existing groups into smaller subgroups) so that the weighted in/out-degree variability of nodes that are in the same subgroup is reduced. The process is as follows. We take two parameters $v_{\text{in}}, v_{\text{out}}$ that define the maximal in- and out-degree variability allowed in the new partition. This means that the new partition has to be such that all the weighted in- and out-degrees (coming from and ending at nodes in all the other groups) of two nodes that are in the same group can differ, at most, by v_{in} and v_{out}, respectively. For this we first compute all the weighted in- and out-degrees of nodes in each group, coming and ending at all the other groups (i.e., for each node we have n in-degrees and n out-degrees, where n is the number of groups in the original partition). Then, for each pair of groups G_ν, G_ρ, we order the in(out)-degrees of nodes in G_ν from (to) G_ρ and we classify these degrees into categories so that the difference between the minimal and maximal degree within each category is smaller than the desired threshold v_{in} (v_{out}) and so that the number of categories is as small as possible. Now we classify all the nodes in G_ν according to these degree categories: two nodes end up in the same subgroup whenever all their degrees have fallen in the same category.

Computing the bifurcation diagrams

To compute the bifurcation diagram of a system, we first create a network instantiation or take a network from given data. If the network is binary, the adjacency matrix is converted into a positive matrix by setting all the missing connections to a very small value $\epsilon > 0$. We then vary the overall strength of connections by multiplying all the interaction weights by a common factor d. For each value of d we compute the homogeneous and spectral reductions and we integrate both the original and the reduced dynamics to equilibrium. Once the equilibrium values of the n observables (exact or reduced) are known, we compute their weighted average according to group size:

$$\langle \mathcal{X} \rangle := \frac{1}{N} \sum_{\nu=1}^{n} m_\nu \, \mathcal{X}_\nu . \tag{S85}$$

We also define $\mathcal{K}_\nu$ as the weighted in-degree of observable ν in the reduced system:

$$\mathcal{K}_\nu := \sum_{\rho=1}^{n} \mathcal{W}_{\nu\rho}, \tag{S86}$$

and from it we can compute the average in-degree in the reduced system, weighted by the group size:

$$\langle \mathcal{K} \rangle := \frac{1}{N} \sum_{\nu=1}^{n} m_\nu \, \mathcal{K}_\nu . \tag{S87}$$

We finally generate a diagram that shows the state of the observable average $\langle \mathcal{X} \rangle$ (exact and reduced) at equilibrium as a function of $\langle \mathcal{K} \rangle$.

Examples of node dynamics

Neuronal dynamics. We take as an example of neuronal dynamics Hopfield's continuous model [1]. Each node in the network represents a neuron that receives and projects inputs to the other neurons via synaptic connections. The node activity x_i represents the mean membrane potential of neuron i and evolves according to

$$\dot{x}_i = -x_i + \sum_{j=1}^{N} w_{ij} \, g(x_j), \tag{S88}$$

where g is a sigmoid function of one variable only which transforms the potential x_j of the presynaptic neuron j into its output $g(x_j)$ (for example, its firing rate). Thus, in this case we have $g(x, y) = g(y)$. We specifically take the function g to be

$$g(y) = \frac{1}{1 + \exp(-\tau(y - \mu))}, \tag{S89}$$

where τ and μ are two parameters that control the maximal slope of g and its location. Hopfield's continuous model is closely related to several well-known models of neuronal activity on networks, such as the Wilson-Cowan and Grossberg models [2, Sec. 6.C] or the firing-rate model [3, p. 360].

Infectious dynamics. The SIS (susceptible-infected-susceptible) model aims at describing the spread of a disease in a network of contacts. Each node can be in two possible states: susceptible or infected. The node state stochastically evolves in time according to the states of the nodes it is in contact with: a susceptible node becomes infected at a rate λ times the number of infected contacts and an infected node becomes susceptible again at a constant rate of 1. It is possible to define a mean-field version of the model that specifies the temporal evolution of the probability x_i of node i being infected in the contact network [4, Sec. V.A.2]:

$$\dot{x}_i = -x_i + \gamma(1 - x_i) \sum_{j=1}^{N} w_{ij} \, x_j , \tag{S90}$$

where $\gamma \geq 0$ is the normalized infection rate.

Ecological dynamics. We consider a network of interacting species in a given ecosystem. If x_i represents the abundance of species i, the evolution of the species' abundances can be modeled by

$$\dot{x}_i = B + x_i \left(1 - \frac{x_i}{K}\right) \left(\frac{x_i}{C} - 1\right) + \sum_{j=1}^{N} w_{ij} \frac{x_i x_j}{D + E x_i + H x_j}, \tag{S91}$$

where B is a constant migration rate, $K > 0$ is a carrying capacity and $C > 0$ is the minimum abundance of species i for it to grow [5]. The parameters D, E, H shape the inter-species coupling dynamics. We assume that the adjacency matrix is positive, so the dynamics is that of a mutualistic network.

Additional figures

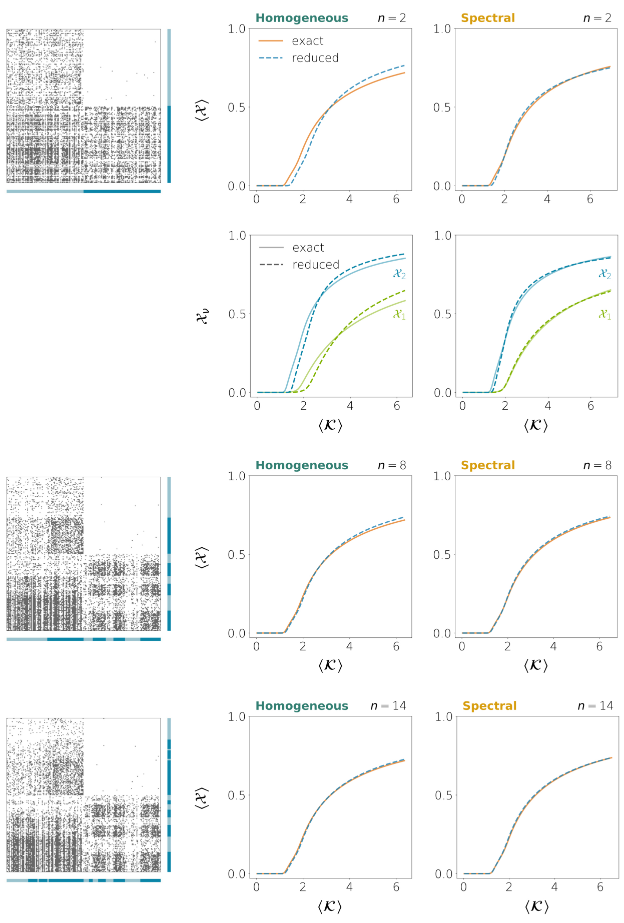

Fig. S2. Exact versus reduced bifurcation diagrams for a heterogeneous directed network and infectious dynamics (Eq. (S90) with $\gamma = 1$). The network has $N = 200$ nodes and 2 communities of the same size and with mean connection densities $p_{11} = 0.2$, $p_{12} = 0.001$, $p_{21} = 0.5$, $p_{22} = 0.3$.

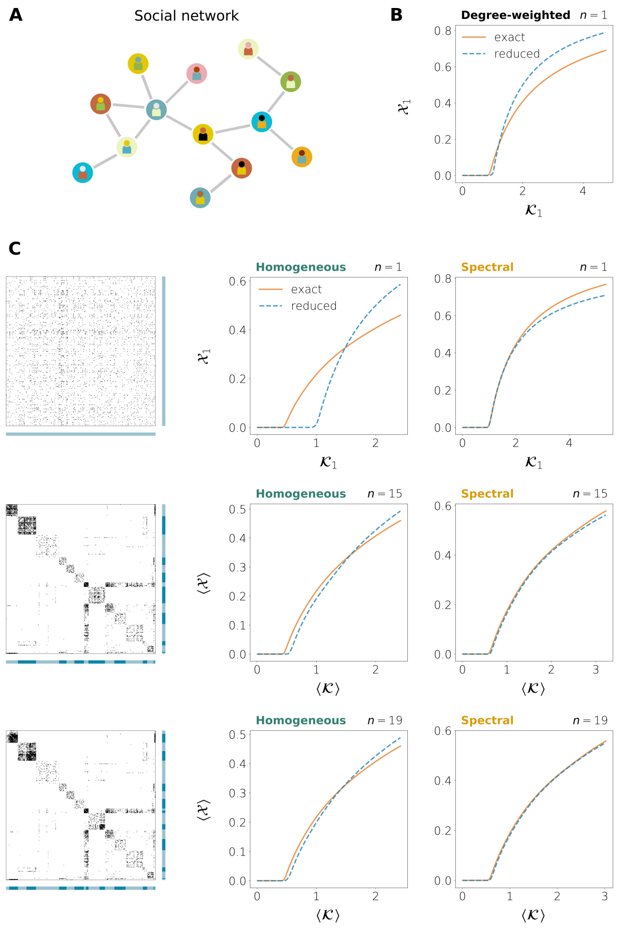

Fig. S3. SIS infectious dynamics (Eq. (S90), $\gamma = 1$) on a social network based on Facebook contacts [6]. The network has $N = 362$ nodes and it is binary and undirected. **A**. Schematics of a social network. **B**. Bifurcation diagram obtained from the degree-based reduction defined in Ref. [7]. **C**. Bifurcation diagrams for the homogeneous and the spectral methods when the whole network is taken as a single group ($n = 1$) and for successive refinements of a partition on $n = 15$ groups.

References

1. J. J. Hopfield. Neurons with graded response have collective computational properties like those of two-state neurons. *Proc. Natl. Acad. Sci. U.S.A.*, 81:3088–3092, 1984. doi:10.1073/pnas.81.10.3088.
2. S. Grossberg. Nonlinear neural networks: Principles, mechanisms, and architectures. *Neural Netw.*, 1:17–61, 1988. doi:10.1016/0893-6080(88)90021-4.
3. T. P. Vogels, K. Rajan, and L. F. Abbott. Neural network dynamics. *Annu. Rev. Neurosci.*, 28:357–376, 2005. doi:10.1146/annurev.neuro.28.061604.135637.
4. R. Pastor-Satorras, C. Castellano, P. Van Mieghem, and A. Vespignani. Epidemic processes in complex networks. *Rev. Mod. Phys.*, 87:925–979, 2015. doi:10.1103/RevModPhys.87.925.

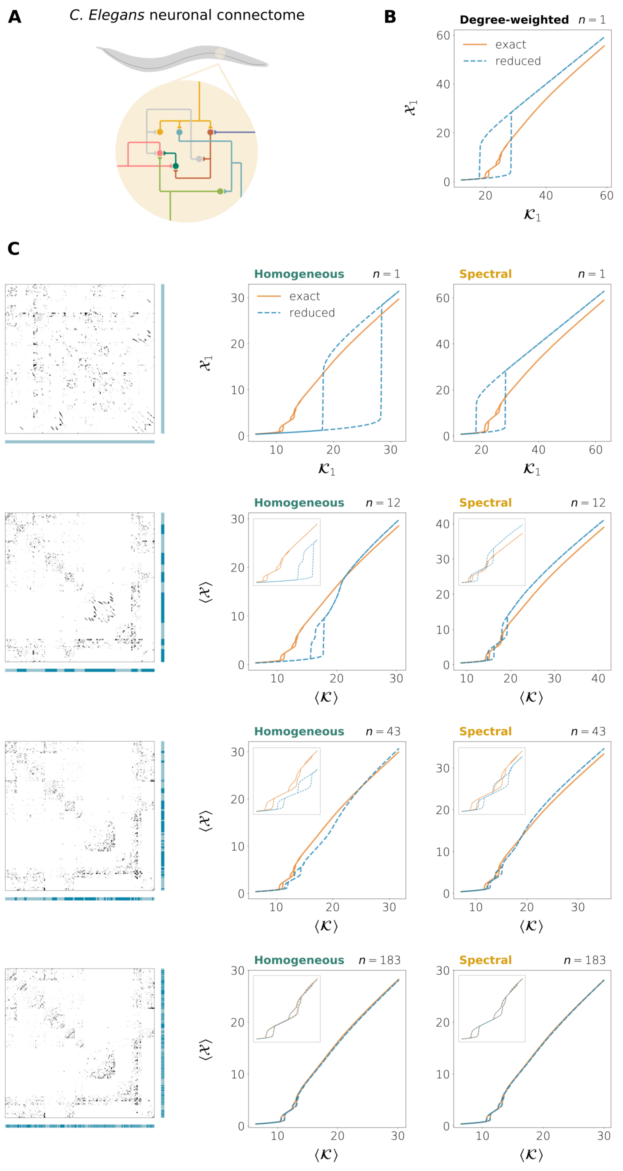

Fig. S4. Neuronal dynamics (Eq. (S88), same parameters as in Fig. 2) on the connectome of the worm *C. Elegans* [8]. The network is weighted and directed and contains $N = 279$ neurons. **A.** Schematics of the *C. Elegans* connectome. **B.** Bifurcation diagram obtained from the degree-based reduction defined in Ref. [7]. **C.** Bifurcation diagrams for the homogeneous and the spectral methods when the whole network is taken as a single group ($n = 1$) and for successive refinements of a partition on $n = 12$ groups.

5. J. N. Holland, D. L. DeAngelis, and J. L. Bronstein. Population Dynamics and Mutualism: Functional Responses of Benefits and Costs. *The American Naturalist*, 159: 231–244, 2002. doi:10.1086/338510.
6. B. F. Maier and D. Brockmann. Cover time for random walks on arbitrary complex networks. *Phys. Rev. E*, 96: 042307, 2017. doi:10.1103/PhysRevE.96.042307.
7. J. Gao, B. Barzel, and A.-L. Barabási. Universal resilience patterns in complex networks. *Nature*, 530:307–312, 2016. doi:10.1038/nature16948.
8. B. L. Chen, D. H. Hall, and D. B. Chklovskii. Wiring optimization can relate neuronal structure and function. *Proc. Natl. Acad. Sci. U.S.A.*, 103:4723–4728, 2006. doi:10.1073/pnas.0506806103.
